# Zero- to One-Dimensional Transformation in a Highly Porous Metal–Organic Framework to Enhance Physicochemical Properties

**DOI:** 10.1021/jacs.5c03967

**Published:** 2025-05-07

**Authors:** Enhui Jiang, Daisong Chen, Zhuoliang Ying, Jiaming Zhou, Artit Jarusarunchai, Xinyu Zhang, Chenxi Xiong, Keunhong Jeong, Dong-Myeong Shin, Jin Shang, Seungkyu Lee

**Affiliations:** † Department of Chemistry, 25809 The University of Hong Kong, Hong Kong, SAR, China; ‡ School of Energy and Environment, 53025 City University of Hong Kong, Hong Kong, SAR, China; ⊥ Department of Mechanical Engineering, 25809 The University of Hong Kong, Hong Kong, SAR, China; ▽ Department of Physics and Chemistry, 65767 Korea Military Academy, Seoul 01805, Republic of Korea

## Abstract

The dynamic behaviors of metal–organic frameworks (MOFs) continue to expand the accessible architectures and properties within this material class. However, the dynamic behaviors that can be studied in MOFs are limited to the transitions, preserving their high crystallinity. For this reason, their significant structural changes involving coordination bond breakage and rearrangement remain largely underexplored. Herein, we report a three-step single-crystal-to-single-crystal (SCSC) phase transition in a new cerium-based MOF, HKU-9 [Ce_2_PET­(DMF)_2_(H_2_O)_2_], transforming zero-dimensional (0D) secondary building units (SBUs) into one-dimensional (1D) chain SBUs in HKU-90 [Ce_2_(μ-H_2_O)­PET­(H_2_O)_2_]. Single-crystal X-ray diffraction studies unambiguously delineate the structural evolution at each stage of this multistep transition, revealing multiple coordination bond dissociations/associations and a significant lattice contraction—all while preserving single-crystal integrity. This dimensional transformation endows HKU-90 with enhanced chemical stability (pH 1–10) and a record-high Brunauer–Emmett–Teller (BET) surface area of 2660 m^2^ g^–1^ among reported Ce-based MOFs. Further, HKU-90 exhibits exceptional gas sorption performance, with one of the highest reported C_2_H_2_ storage capacities (184 cc g^–1^ at 273 K, 1 bar) and outstanding C_2_H_2_/CO_2_ selectivity (2.16) under these conditions. Notably, the formation of 1D chain SBUs, a structural motif found in many high-performance MOFs, highlights the potential of using the solid-state fusion of multinuclear metal clusters to tailor the properties of the framework.

## Graphical abstract

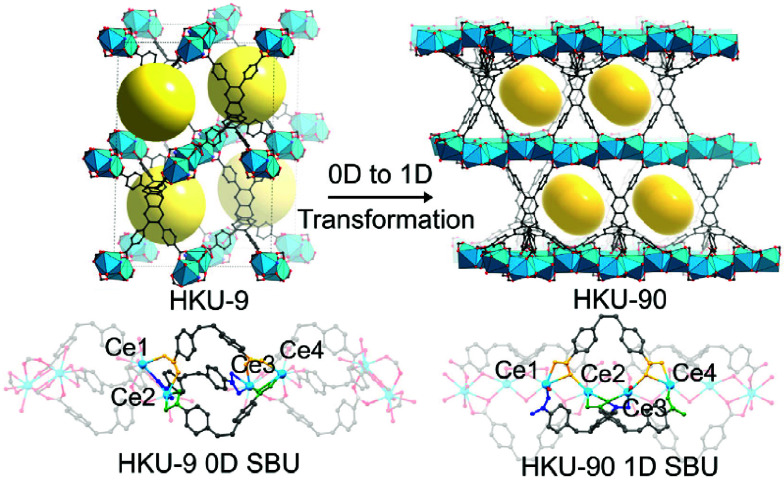

Metal–organic frameworks (MOFs) are a versatile class of crystalline porous materials characterized by their structural diversity and tunable properties, enabling a wide range of design possibilities.^1–4^ This versatility has driven extensive exploration of MOFs in applications such as gas storage and separation, batteries, and biomedical uses.^5–8^

The scope of MOF diversity can be further expanded by considering their dynamic structural behaviors.^9–11^ Phase transitions in MOFs, which encompass a range of structural transformations from breathing and expansion/contraction to more dramatic changes involving decomposition and recrystallization, offer pathways to access new frameworks that are challenging to synthesize directly through *de novo* methods.^12–14^ In addition, these transitions provide opportunities to modulate key properties, such as gas sorption/desorption and photoluminescence, thereby broadening the functional versatility of MOFs.^15–17^

Single-crystal X-ray diffraction (SXRD) studies on single-crystal-to-single-crystal (SCSC) phase transitions provide precise structural information on the initial and final phases at the atomic level.^18–20^ These studies could be uniquely valuable for identifying a continuous structural trajectory between the phases because the single-crystal nature minimizes the likelihood of complete decomposition and recrystallization.^21^ As a result, the structural correlation between the initial and final phases allows for high-confidence investigations into the transition mechanisms, offering insights into the fundamental dynamic behaviors of the MOFs.

However, multiple steps of single-crystalline phase transitions with each involving dramatic dissociation and reassociation of coordination bonds are rarely observed in MOFs.^22,23^ Since MOFs are extended structures, such transitions require highly coordinated structural rearrangements across the crystal lattice, which are difficult to achieve without disrupting the long-range order of the single crystals.^24,25^ The disorder is more pronounced in highly porous structures, where the large void spaces allow significant dislocations of the building units during the transitions.^26^ In dense structures, such large deviations are constrained. These limit studying dynamic bond breaking and formation in MOFs, as it restricts the range of structural transformations that can be examined using single crystals.

Herein, we report unusual dramatic SCSC phase transitions in a new MOF, HKU-9 [Ce_2_PET­(DMF)_2_(H_2_O)_2_], where HKU stands for The University of Hong Kong, and PET represents peripheral extended triptycene, which highlights the solid-state dimensional transformation of zero-dimensional (0D) secondary building units (SBUs) into 1D chain SBUs of HKU-90 [Ce_2_(μ-H_2_O)­PET­(H_2_O)_2_]. The overall transition was analyzed into three steps with the SXRD technique. The SCSC phase transitions involve multiple metal–ligand bond dissociations and formations, coordination environment rearrangements, and a significant volume contraction, all while preserving single-crystalline structural integrity. The results show that 1D SBUs of MOFs can be synthesized by the fusion of predefined multiple metal clusters in the solid state.

Notably, HKU-90 exhibits enhanced chemical stability and a record high Brunauer–Emmett–Teller (BET) surface area (2660 m^2^/g) among the reported Ce-based MOFs.^27,28^ In addition, HKU-90 shows a C_2_H_2_ storage capacity of 184 cc/g at 273 K and 1 bar—one of the highest among reported MOFs—and a C_2_H_2_/CO_2_ selectivity of 2.16, also among the highest under the same conditions.^29–31^ These findings highlight the functional benefits of phase transitions in MOFs.

This unusual dimension-changing phase transition provides a foundation for further exploration of 1D SBU formation across solid- and solution-phase systems and offers potential strategies to study MOF formation mechanisms from solid state phase transitions.

HKU-9 was synthesized via a solvothermal method. Ammonium cerium­(IV) nitrate, (NH_4_)_2_Ce­(NO_3_)_6_, and the H_6_PET linker were combined in a 6:1 molar ratio in *N,N*-dimethylformamide (DMF) within a scintillation vial. The mixture was subsequently heated to 100 °C in a preheated oven. After 24 h, block-shaped crystals of HKU-9 were observed at the bottom of the vial (see Supporting Information (SI) section S1).^32,33^ The as-synthesized crystals were used to conduct the SXRD experiment. The refined structure in the *C*2/*c* space group is shown in Figure 1.^34–36^ The SBUs, [Ce_2_(−COO)_6_DMF_2_(H_2_O)_2_], of as-synthesized HKU-9 are 0D metal clusters consisting of two Ce (III) ions^37^ coordinated by six carboxylate groups from the PET linkers, two DMF molecules, and two water molecules (Figure 1).

**Figure 1:**
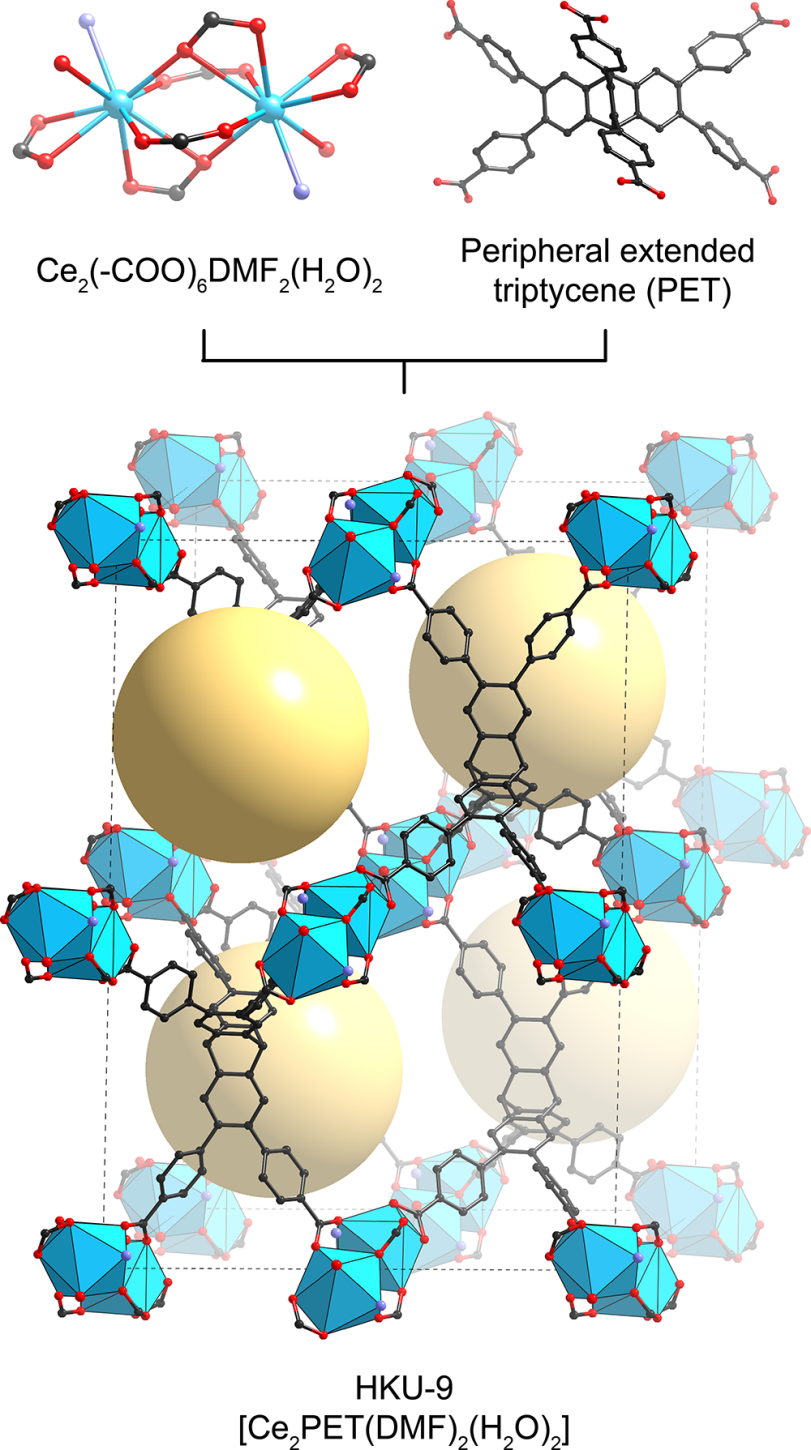
The structures of the building units and as-synthesized HKU-9. Four potential open metal sites in an SBU are coordinated by two DMF molecules and two water molecules. The coordinated oxygen atoms are indicated with pale purple for DMF and red for water. The structure of the as-synthesized HKU-9 is shown in a polyhedral model. Yellow balls indicate the approximate size and location of the pores.

The two Ce ions are bridged by four carboxylates: two in a symmetric monodentate bridging mode and the other two in an asymmetric bidentate bridging mode. The remaining two carboxylates chelate each Ce ion, contributing to the elongated octahedral geometry of the SBU. Each Ce ion has two potential open metal sites, occupied by DMF and water molecules. The SBUs are linked by six trigonal prismatic PET linkers, where each linker connects to six SBUs to form an extended structure with **nia** 3D net topology.

SCSC phase transitions were observed during a typical guest-removal activation procedure for MOFs: washing with DMF, solvent exchange with acetone, and degassing. Powder X-ray diffraction (PXRD) patterns were collected after each step to monitor structural changes (Figure 2). Intriguingly, the PXRD patterns are significantly different at each stage, with changes that cannot be explained by simple peak shifts, implying that substantial phase transitions occur during the process.

**Figure 2:**
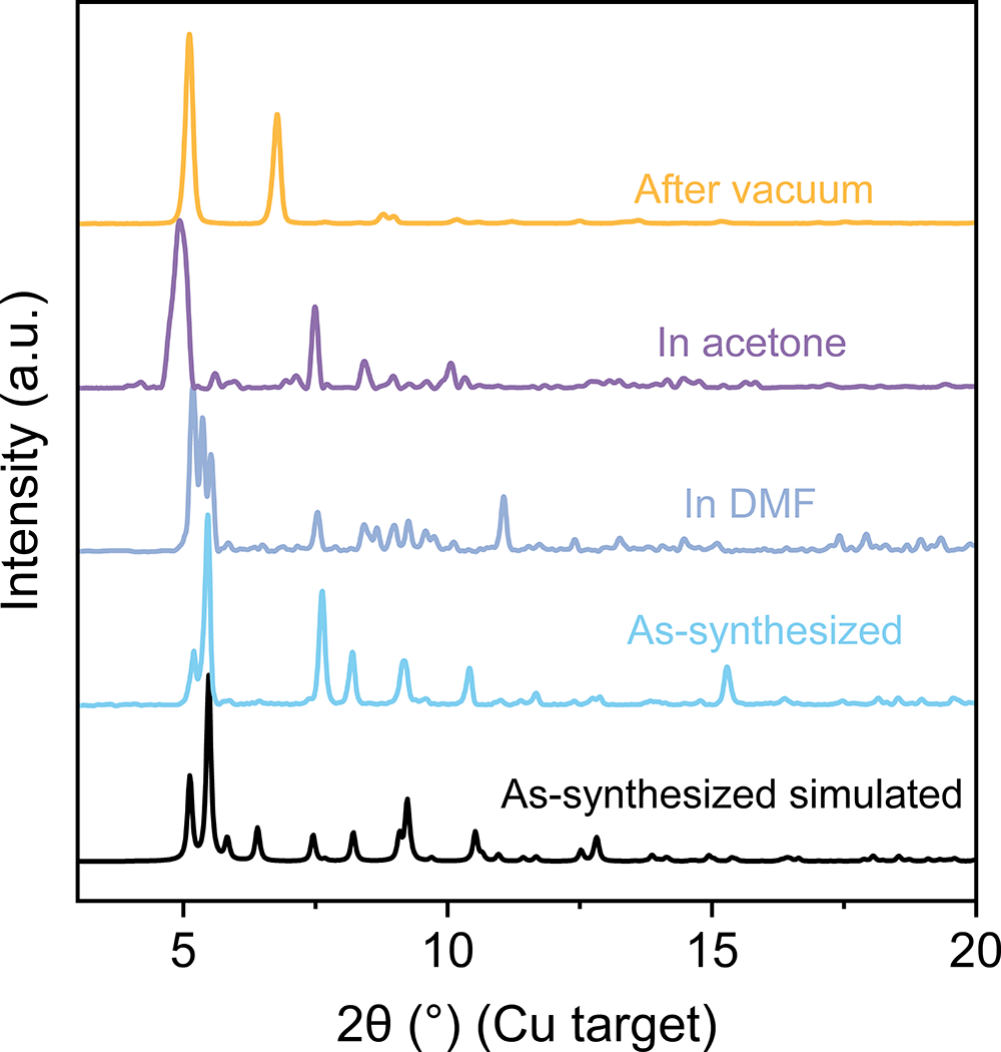
PXRD pattern comparison of HKU-9 crystals after each step of the phase transition.

The structures of the crystals from each stage were analyzed using SXRD. All samples were highly crystalline with diffraction data collected to a resolution of 0.80 Å or higher. The refined structures are shown in Figure 3. In the as-synthesized structure, the closest distance between Ce atoms in adjacent SBUs is 10.77 Å, which is too far to be bridged. Upon replacing the initial neutral ligands (H_2_O and DMF) with fresh DMF, the Ce–Ce distance decreased to 9.53 Å, and further solvent exchange with acetone reduced it to 8.29 Å (Figure 3b).

**Figure 3:**
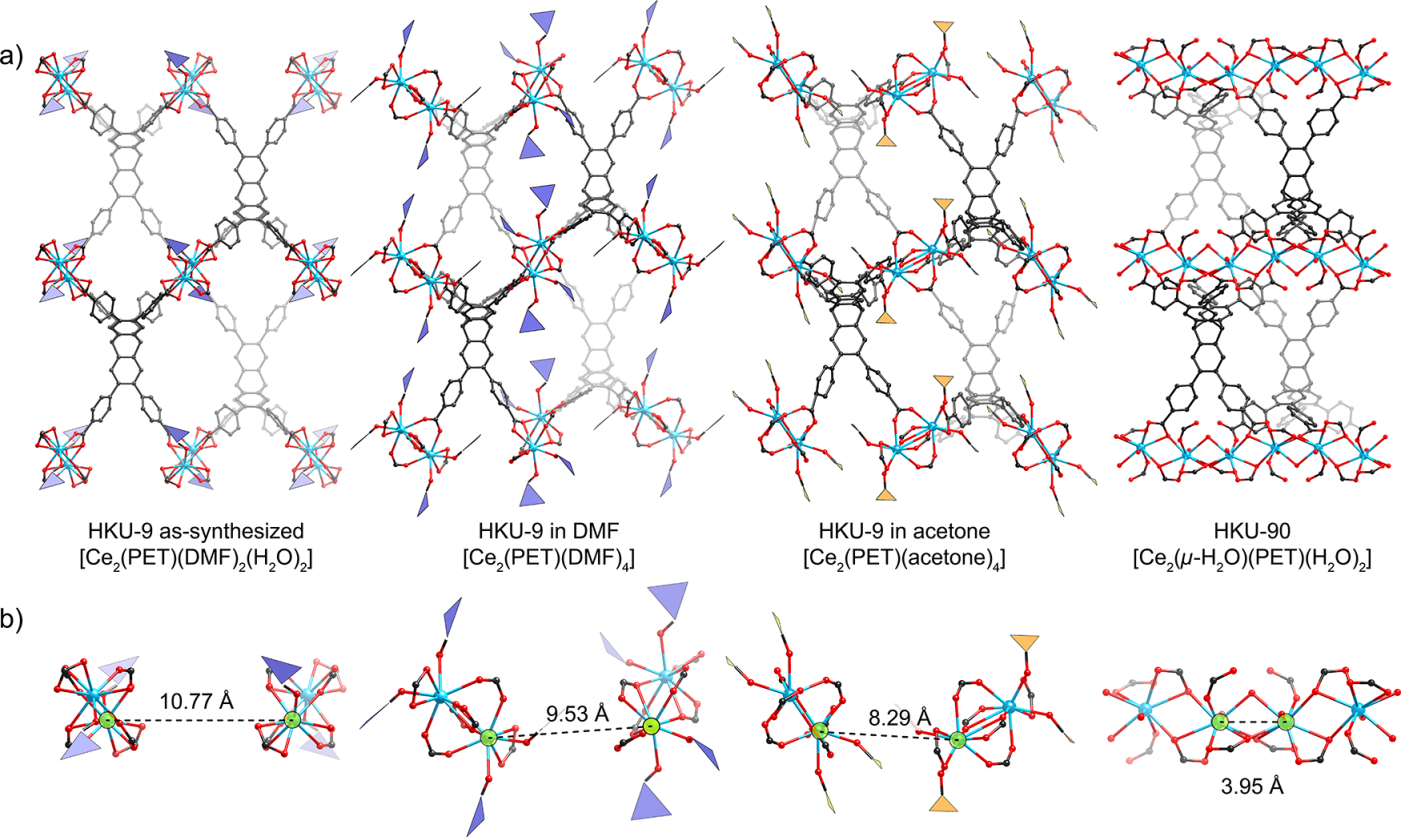
Refined crystal structures of HKU-9 after each step of the phase transition. a) Structures are shown as ball-and-stick models, with DMF and acetone molecules coordinated to Ce ions indicated in purple and yellow polygons, respectively, for clarity. b) Two adjacent SBUs from each structure are displayed below, with distances between the closest Ce–Ce ions on different SBUs indicated.

Interestingly, during these transitions, the coordination modes of the six carboxylates appear to interconvert, such as from monodentate bridging to asymmetric bidentate bridging mode, while the overall initial combination of two monodentate bridging, two bidentate bridging, and two chelating configurations is preserved. This rearrangement is likely driven by the formation of thermodynamically stable configurations, which are predominantly influenced by the neutral ligands. The varying sizes of the neutral ligands induce distinct van der Waals interactions and steric effects with adjacent coordinating carboxylates and phenyl rings. This suggests that the relatively weak Ce–O interactions^4^ facilitate bond dissociation and association and ligand rearrangements within the coordination sphere, enabling the observed structural transitions.

Finally, the adjacent SBUs of HKU-9-acetone are bridged by μ-H_2_O and carboxylate ligands, forming HKU-90 when the sample was activated under a vacuum and exposed to air (Figures 3a and 4b). We also observed the completion of the transition by simply drying HKU-9-acetone in air in 5 min. The structure of HKU-90 with 1D chain SBUs was determined with 0.70 Å resolution SXRD data. The structure was refined in the *C*2/*c* space group with unit cell parameters, *a* = 21.0 Å, *b* = 34.1 Å, *c* = 16.3 Å, and β = 103.7° (Figures 3a and 4c). In the structure, the distance between Ce and Ce, bridged by the μ-H_2_O and two carboxylates, is 3.95 Å. The reactive open metal sites generated by removing acetone under vacuum could react with water to bridge the SBUs.^38–41^ The other open metal site in the Ce ion is coordinated with a water molecule in HKU-90 (Figures 3 and 4b). Density functional theory calculations confirm the spontaneous and exothermic nature of the transformation, primarily driven by a significant decrease in enthalpy, with a mild decrease in entropy associated with the more ordered linear SBUs in HKU-90. Computational analyses further highlight the crucial stabilizing role of electrostatic interactions between bridging water molecules and cerium atoms, supported by detailed charge distribution and noncovalent interaction (NCI) mapping (SI Section S4.3).

**Figure 4:**
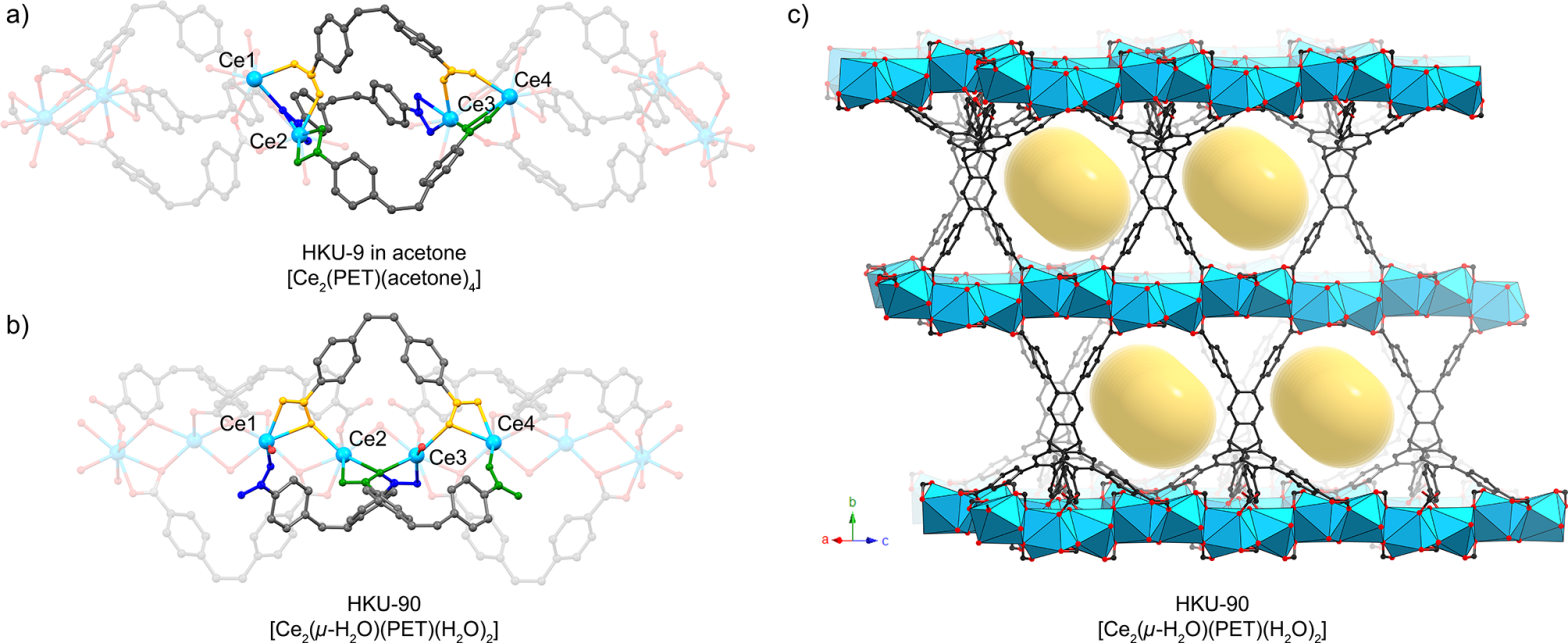
Transformation of the 0D SBUs of HKU-9 into 1D SBUs of HKU-90. a) The refined structure of the SBUs of HKU-9-acetone is shown. The six carboxylate ligands from the three different PET linkers that are linking the adjacent SBUs are emphasized with different colors (yellow, green, and blue). Carboxylates from the same linker are indicated with the same color. Ce ions are numbered from left to right. b) Corresponding carboxylate ligands and Ce ions in HKU-90 are indicated in the same manner. c) HKU-90 structure viewed from the near [101] direction. Yellow cylinders indicate the approximate size and location of newly formed 1D channels after the transformation.

To understand the rearrangement of carboxylates, the SBUs of HKU-9-acetone and HKU-90 are compared in Figure 4a and b. After the transition, significant rearrangements of carboxylates involving coordination bond dissociation and association are observed to accommodate the reduced Ce–Ce distance. The transition can be understood by rearrangements of three pairs of carboxylates from three different PET linkers linking two adjacent SBUs in HKU-9-acetone (Figure 4a). One pair of carboxylates (yellow) coordinates to the SBUs in a monodentate bridging mode. These carboxylates changed to bidentate bridging modes in HKU-90 (Figure 4b). The other two pairs (green and blue) coordinate in a similar manner in HKU-9-acetone and rearrange in a similar manner. In one pair (green), one carboxylate chelates to Ce2, and the other carboxylate bridges Ce3 and Ce4 in a bidentate bridging mode. After the transition, the chelating carboxylate in HKU-9-acetone becomes a bridge between Ce2 and Ce3 in a bidentate bridging mode in HKU-90. The other carboxylate changed to coordinate to Ce4 in a monodentate dangling mode. All these changes, accompanying serious composition change, volume contraction, and coordination bond rearrangements, occurred in a concerted manner across the extended framework of the single crystals, preserving the high crystallinity, as evidenced by the high-resolution diffraction data (Table S4). The transformation of SBUs from 0D to 1D was irreversible upon soaking HKU-90 in acetone and DMF (Figure S11).

The SCSC transition improved several practical aspects of the MOF (Figure 5). While as-synthesized HKU-9 is not stable in water and cannot be activated due to pore collapse, activated HKU-90 showed a high chemical stability from pH 1 to pH 10 in aqueous solution for 3 days (Figure 5a). Moreover, HKU-90 showed the highest surface area (2660 m^2^ g^–1^) among reported Ce-based MOFs (Figure 5b and Table S6).^42–53^ Gas adsorption experiments with C_2_H_2_, CO_2_, CH_4_, N_2_O, and N_2_ (Figure 5c) reveal that HKU-90 achieves one of the highest C_2_H_2_ storage capacities: 184 cc/g (8.22 mmol/g) at 1 bar and 273 K (Table S7) while displaying a relatively low CO_2_ uptake of 85.1 cc/g (3.80 mmol/g) under the same conditions. Although the C_2_H_2_/CO_2_ selectivity of 2.16 may not appear impeccable, it is among the highest reported for MOFs under these conditions (Table S7).^54–61^ The nearly linear adsorption isotherms for C_2_H_2_ and CO_2_, along with the relatively low zero-loading heats of adsorption (24 kJ/mol for C_2_H_2_ and 19 kJ/mol for CO_2_), suggest that the open metal sites do not significantly contribute to the observed high uptake and selectivity (SI Section S4.4). Instead, the high C_2_H_2_ uptake is likely attributed to the high surface area of the MOF, while the selectivity probably arises from the stronger interaction of C_2_H_2_ due to its higher quadrupole moment compared to that of CO_2_ and their multiple weak interactions with the framework. Given that C_2_H_2_ is a critical feedstock for various industrial processes and that CO_2_ impurity can lead to undesired side products—compounded by the challenges of separating CO_2_ due to its similar molecular size and physicochemical properties^27^—the enhanced chemical stability, high C_2_H_2_ storage capacity, and excellent selectivity of HKU-90 offer promising potential for developing energy-efficient gas separation systems, warranting further investigation and optimization.

**Figure 5:**
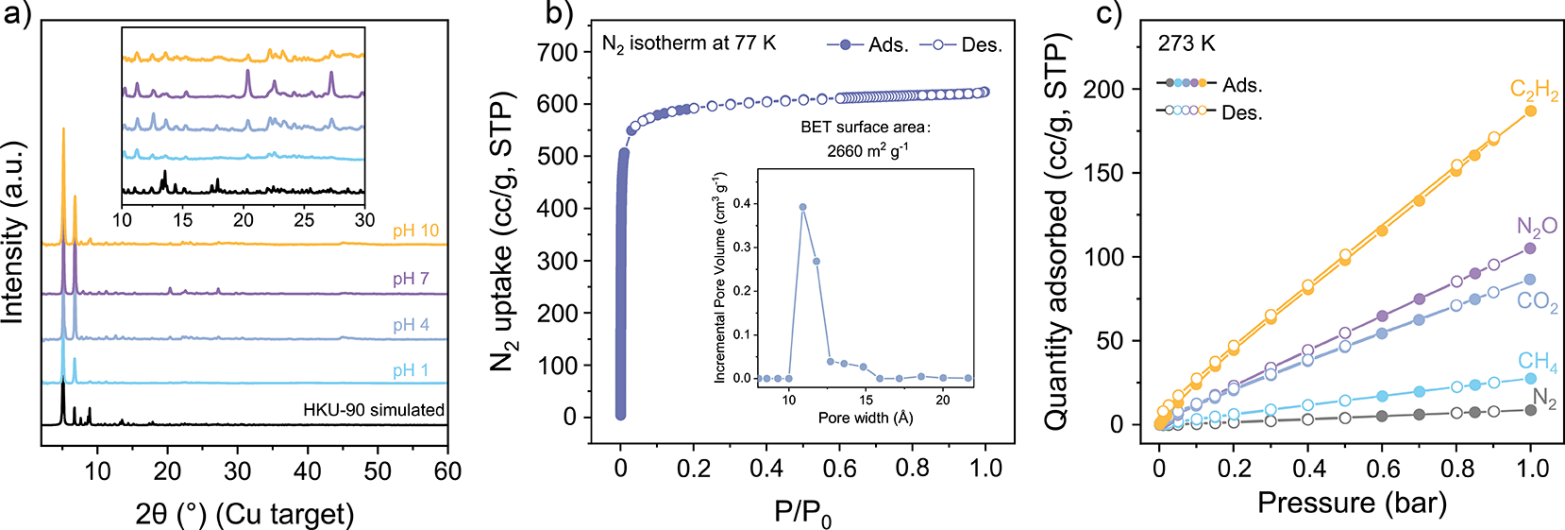
Enhanced chemical stability and adsorption properties of HKU-90. a) PXRD patterns obtained after water stability test in various pH ranges. b) N_2_ isotherm measured at 77 K. c) Adsorption isotherms of various gases on HKU-90 at 273 K.

This study showed an unprecedentedly significant SCSC phase transition in Ce-based MOFs, HKU-9, transforming its 0D SBUs into 1D chain SBUs in HKU-90. The phenomenon expands the scope of significant structural change, which can be studied in MOFs in sub-angstrom resolution. Further exploration of dimension-changing phase transitions in MOFs can potentially offer a strategic approach to designing materials with tailored properties for energy-efficient gas separations and other advanced applications. In particular, 1D chain SBUs are structural motifs central to many benchmark MOFs.^62^ However, systematic and controlled variations of SBUs remain limited. Our findings suggest that stepwise synthesis toward 1D SBUs is achievable, which might enable a meaningful yet limited multimetal sequence along the chain and site-specific functionalization via bridging ligands, rather than the μ-H_2_O groups.

## Supplementary Material



## Notes

Information regarding the synthesis and characterization of the reported MOF, including SXRD experimental conditions and structure refinement procedure, quantum chemistry calculations, and various gas adsorption measurements (PDF)
