# Supplementary material for: Zero- to One-Dimensional Transformation in a Highly Porous Metal–Organic Framework to Enhance Physicochemical Properties
Source: J Am Chem Soc. 2025 May 7;147(20):16766–72. doi: 10.1021/jacs.5c03967 (PMC12100723; doi:10.1021/jacs.5c03967)
Supplement: Supplementary file 1 [file ja5c03967_si_001.pdf]

Supporting Information

Zero- to One-Dimensional Transformation in a Highly Porous Metal–Organic Framework to Enhance Physicochemical Properties

Enhui Jiang,[†,§] Daisong Chen,[‡,§] Zhuoliang Ying,[†] Jiaming Zhou,[⊥] Artit Jarusarunchai,[†] Xinyu Zhang,[†] Chenxi Xiong,[†] Keunhong Jeong,[▽] Dong-Myeong Shin,[⊥] Jin Shang,[‡,*] Seungkyu Lee[†,*]

[†]Department of Chemistry, The University of Hong Kong, Hong Kong SAR, China

[‡]School of Energy and Environment, City University of Hong Kong, Hong Kong SAR, China

[⊥]Department of Mechanical Engineering, The University of Hong Kong, Hong Kong SAR, China

[▽]Department of Physics and Chemistry, Korea Military Academy, Seoul, 01805, Republic of Korea

[*] Corresponding to jinshang@cityu.edu.hk and skchem@hku.hk

Table of contents

Section S1.

Section S1.1. Materials

Section S1.2. Instrumentation

Section S1.3. Synthesis of linker and MOFs

Section S2.

Section S2.1. Powder X-ray diffraction patterns

Section S2.2. Reversibility test of phase transition from HKU-9 to HKU-90

Section S2.3. Optical microscope images

Section S2.4. X-ray photoelectron spectroscopy (XPS)

Section S2.5. Thermogravimetric Analysis (TGA)

Section S2.6. Water stability test of HKU-9

Section S3.

Section S3.1. Single crystal X-ray diffraction experimental conditions

Section S3.2. Single crystal data and structure refinement

Section S4.

Section S4.1. N_2 isotherm measurements

Section S4.2. Various gas adsorption measurements

Section S4.3. DFT calculations for the phase transition

Section S4.4. Heat of adsorption study

References

Figure S1-S25, Table S1-S7

Section S1.

Section S1.1. Materials

Ammonium cerium (IV) nitrate [$(NH_4)_2Ce(NO_3)_6$, 99.99 %], triptycene (purity ≥ 98 %), iron powder (purity ≥ 99 %), (4-ethoxycarbonylphenyl) boronic acid (purity ≥ 98 %), potassium carbonate (K_2CO_3, 99 %), tetrakis(triphenylphosphine)palladium ($Pd(PPh_3)_4$) (purity ≥ 99 %), sodium hydroxide (NaOH) (purity ≥ 98 %), N,N-dimethylformamide (DMF, 99.9%, extra dry, with molecular sieves, water ≤ 30 ppm), were purchased form Energy Chemical Co. Ltd. Bromine (purity ≥ 99.8 %) was purchased form Acros Organics. Magnesium sulfate ($MgSO_4$, anhydrous, 99 %) was purchased from 3A Materials. Chloroform (99 %, GR), dichloromethane (99%, GR), methanol (99 %, GR), hexane (95%, GR), acetone (99.7%, HPLC), and acetic acid (99%, GR) were purchased form Duksan Pure Chemicals Co. Ltd. Hydrochloric acid (HCl, 36%, AR), dioxane (99%, AR), and tetrahydrofuran (THF, 99%, AR) were purchased form RCI Labscan Group Co. Ltd. All chemicals were used as received without further purification.

Section S1.2. Instrumentation

Single-crystal X-ray diffraction (SXRD) data was collected using synchrotron radiation in beamline BL17B1 and BL03HB at Shanghai Synchrotron Radiation Facility (SSRF, China). Beamline BL17B1 and the in-house stations are equipped with Rayonix MX300 detector. In the BL03HB beamline, the X-rays were focused with a toroidal mirror (beam size: 150 × 80 μm^2), and a channel-cut Si (111) monochromator with an energy range of 5-20 keV was used as a monochromator. N_2 adsorption isotherms were recorded on Quantachrome quadrasorb volumetric gas adsorption analyzer. The optical images of as-synthesized, solvent-exchanged, and activated images of HKU-9 and 90 were recorded on a Leica M165 C microscope equipped with a flexacam C3 camera. The powder X-ray diffraction (PXRD) analysis was performed on Rigaku MiniFlex600 X-Ray Diffractometer using Cu metal target radiation source at 40 kV and 15 mA. The samples were mounted on a zero-background sample holder and scanned over the angular range from 2° to 60° (2θ) with a step size of 0.01 at room temperature. Nuclear Magnetic Resonance (NMR) spectra were recorded on Ascend 500 MHz with frequencies of 500 MHz for ^1H and 126 MHz for ^{13}C at room temperature. Thermogravimetric Analysis (TGA) was measured on TA instrument Q50 under N_2 condition. The X-ray photoelectron spectroscopy (XPS) was performed to detect the elemental composition by a Thermo ESCALAB 250X analytical system (USA). Various gas adsorption isotherms were measured on a Micromeritics 3Flex Adsorption Analyzer.

Section S1.3.

Synthesis of 2, 3, 6, 7, 14, 15-hexabromotriptycene

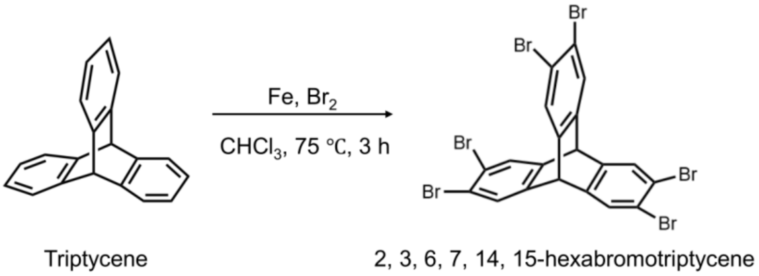

Scheme S1. Synthesis of 2, 3, 6, 7, 14, 15-hexabromotriptycene.

2, 3, 6, 7, 14, 15-hexabromotriptycene was synthesized based on the reported literature[1] with slight modification. A solution of triptycene (2.00 g, 7.8 mmol) and iron powder (67.2 mg, 1.20 mmol) in chloroform (270 mL) was prepared in a reaction flask. A solution of bromine (2.55 mL, 0.100 mmol) in chloroform (70 mL) was added to the flask. The reaction mixture was heated under reflux at 75 °C for 3 h. Flash column chromatography was carried out before the solvent was removed under reduced pressure. The resultant crude product was triturated from dichloromethane and hexane. The precipitate was filtered and washed with methanol several times to give the desired 2, 3, 6, 7, 14, 15-hexabromotriptycene as a white solid: (4.95 g, 6.83 mmol, 88%); ^{1}H NMR (500 MHz, $CDCl_3$): δ (ppm) 7.62 (d, J = 2.7 Hz, 6H, Ar-H), 5.24 (s, 2H, Csp^3-H). ^{13}C NMR (126 MHz, $CDCl_3$), δ = 144.09, 129.22, 121.92, 51.24.

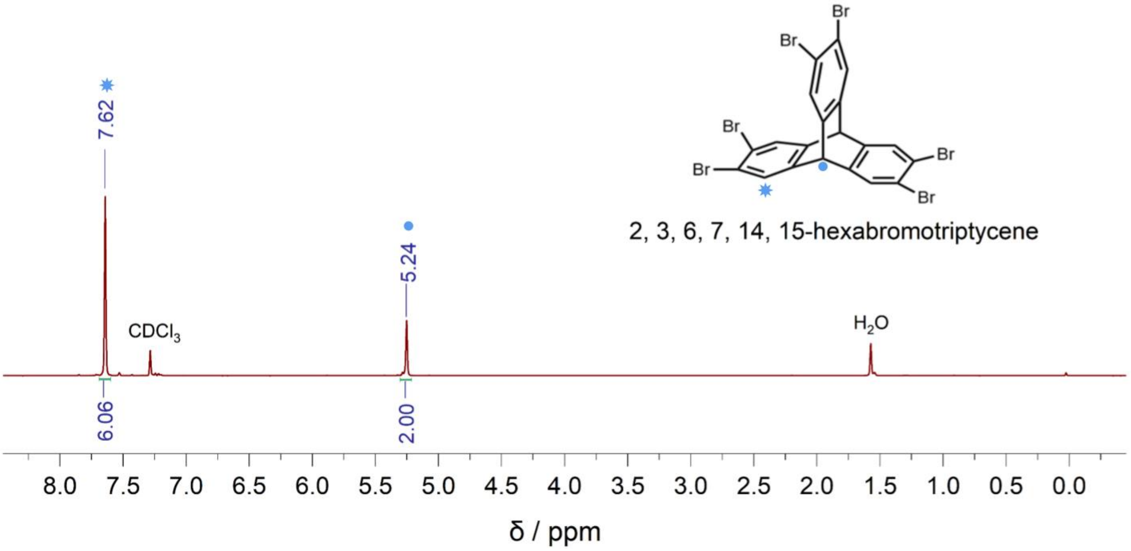

Figure S1. ^{1}H NMR Spectrum (500 MHz, $CDCl_3$, 298 K) of 2, 3, 6, 7, 14, 15-hexabromotriptycene.

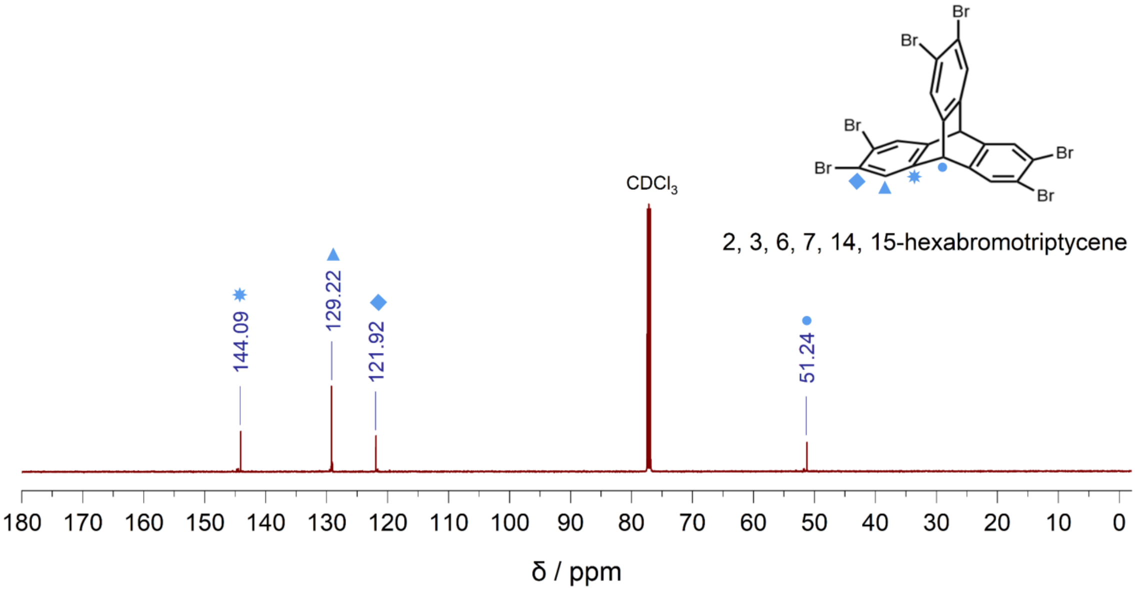

Figure S2. ^{13}C NMR Spectrum (126 MHz, $CDCl_3$, 298 K) of 2, 3, 6, 7, 14, 15-hexabromotriptycene.

Synthesis of Et_6PET

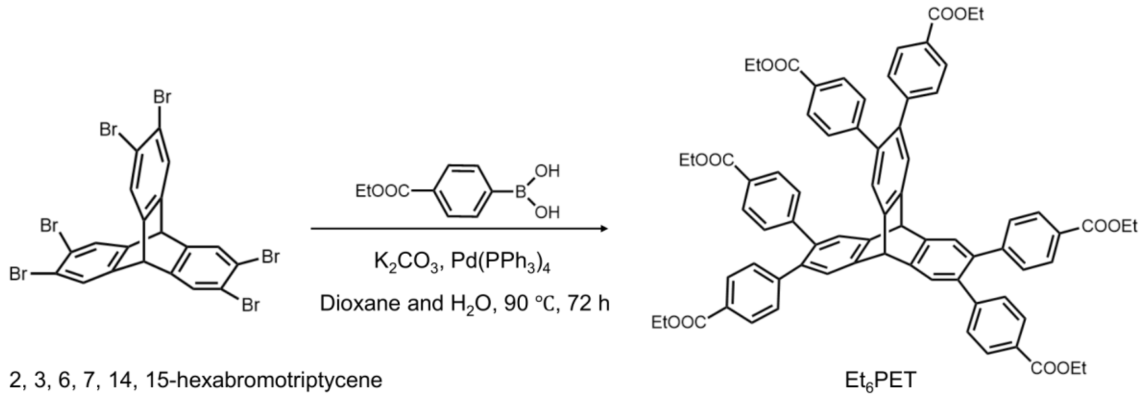

Scheme S2. Synthesis of Et_6PET.

Et_6PET was synthesized based on the reported literature[2] with slight modification. A mixture of 2, 3, 6, 7, 14, 15-hexabromotriptycene (1.50 g, 2.06 mmol), (4-ethoxycarbonylphenyl) boronic acid (3.10 g, 16.00 mmol), K_2CO_3 (2.47 g, 17.8 mmol), and $Pd(PPh_3)_4$ (0.49 g, 0.43 mmol) was evacuated under vacuum, and backfilled with argon in the flask. Afterwards, a degassed mixture of dioxane (100 mL) and water (20 mL) was introduced into the flask. The reaction mixture was heated under reflux at 90 °C for 72 h. The reaction was allowed to cool down to room temperature, and filtered through celite. After removing the solvent under reduced pressure, the crude product was dissolved in dichloromethane (50 mL). The solution was washed by extraction with water. The organic phase was combined and dried over

anhydrous $MgSO_4$. After removing the solvent, the crude solid was purified by column chromatography (SiO_2, 0% to 5% EtOAc in CH_2Cl_2) to give the product as a colorless solid film (1.92 g, 90%); ^{1}H NMR (500 MHz, $CDCl_3$): δ = 7.86 (d, *J* = 8.1 Hz, 12H, Ar-H), 7.58 (s, 6H, Ar-H), 7.14 (d, *J* = 8.4 Hz, 12H, Ar-H), 5.72 (s, 2H, Csp^3-H), 4.34 (q, *J* = 7.1 Hz, 12H, OEt), 1.37 (t, *J* = 7.1 Hz, 18H, OEt). ^{13}C NMR (126 MHz, $CDCl_3$), δ = 166.55, 145.66, 144.58, 137.31, 129.96, 129.47, 128.89, 126.27, 61.12, 53.15, 14.41.

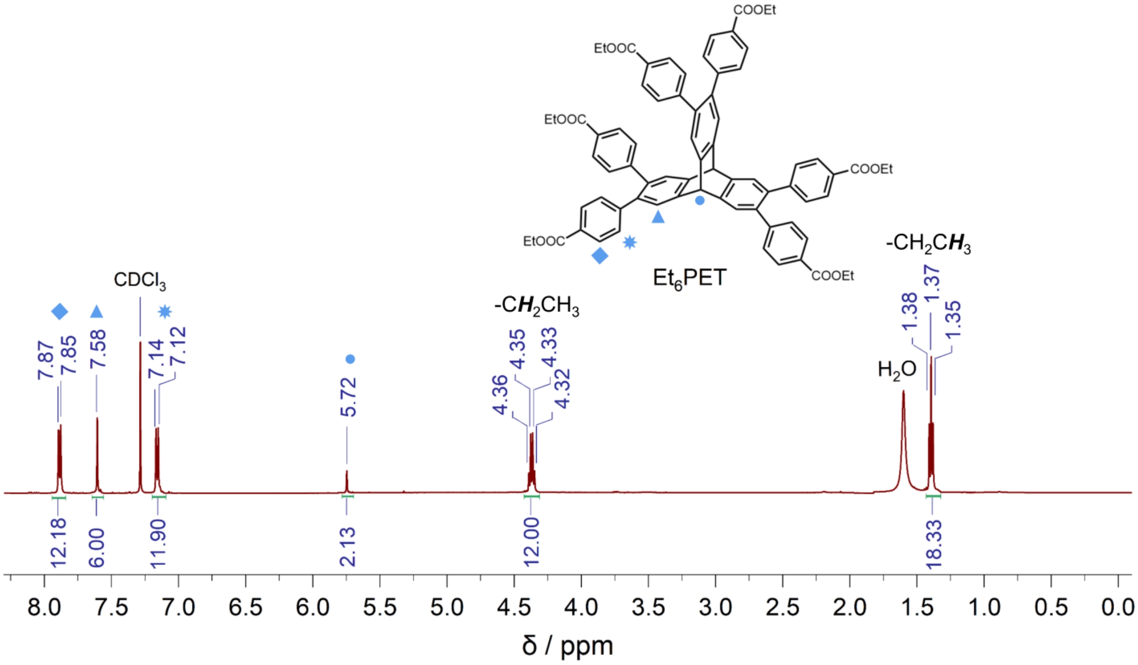

Figure S3. ^{1}H NMR Spectrum (500 MHz, $CDCl_3$, 298 K) of Et_6PET.

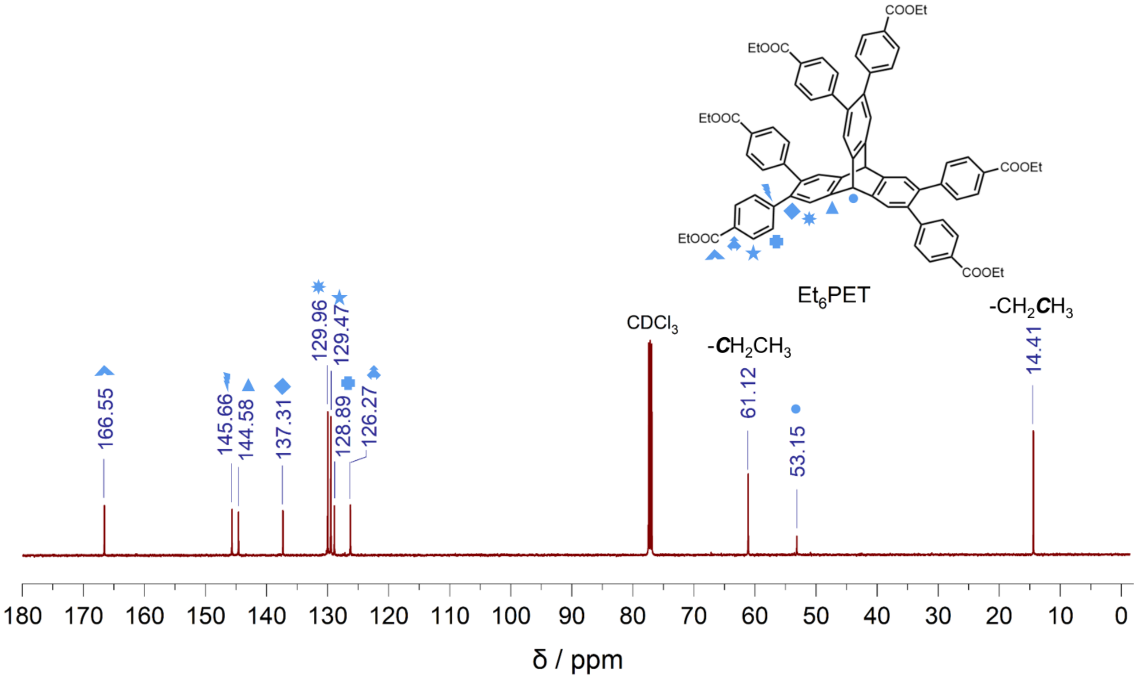

Figure S4. ^{13}C NMR Spectrum (126 MHz, $CDCl_3$, 298 K) of Et_6PET.

Synthesis of H_6PET

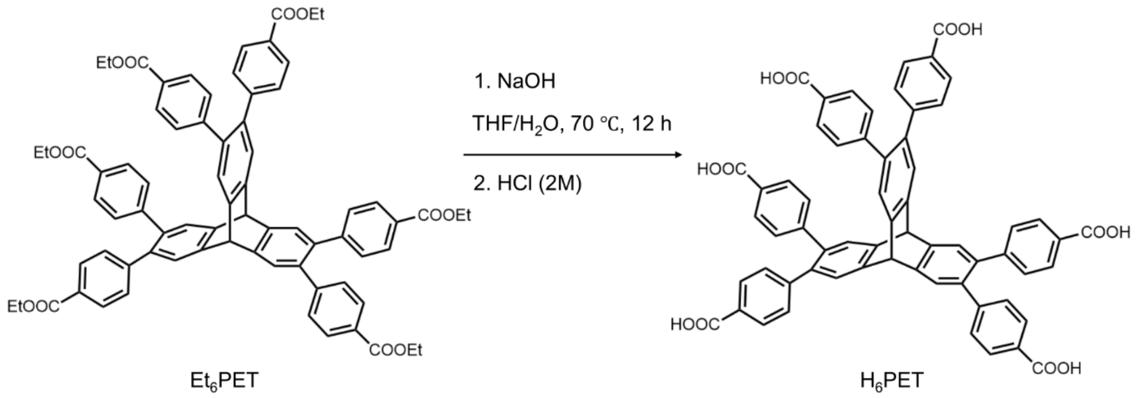

Scheme S3. Synthesis of H_6PET.

Et_6PET (1.30 g, 1.22 mmol) was dissolved in THF (15 mL) in a 100 mL round-bottomed flask equipped with a magnetic stirrer bar. Aqueous NaOH solution (1 M, 20 mL) was added and the resulting mixture was heated at 70 ˚C for 18 h. Upon cooling to room temperature, THF was removed under reduced pressure, and the remaining aqueous solution was acidified with 2 M aqueous HCl solution until pH = 1. The resulting white precipitate was collected by filtration, washed with H_2O (20 mL) and dried under high vacuum to give the product as a white solid (1.41 g, 90%). ^{1}H NMR (500 MHz, DMSO-d_6), δ = 12.92 (s, 6H, COOH), 7.78 (d, J = 8.2 Hz, 12H, Ar-H), 7.66 (s, 6H, Ar-H), 7.19 (d, J = 8.1 Hz, 12H, Ar-H), 6.11 (s, 2H, Csp^{3}-H). $_{13}$C NMR (126 MHz, DMSO-d_6), δ = 167.06, 145.19, 144.76, 136.33, 129.83, 129.09, 129.00, 126.06, 51.32.

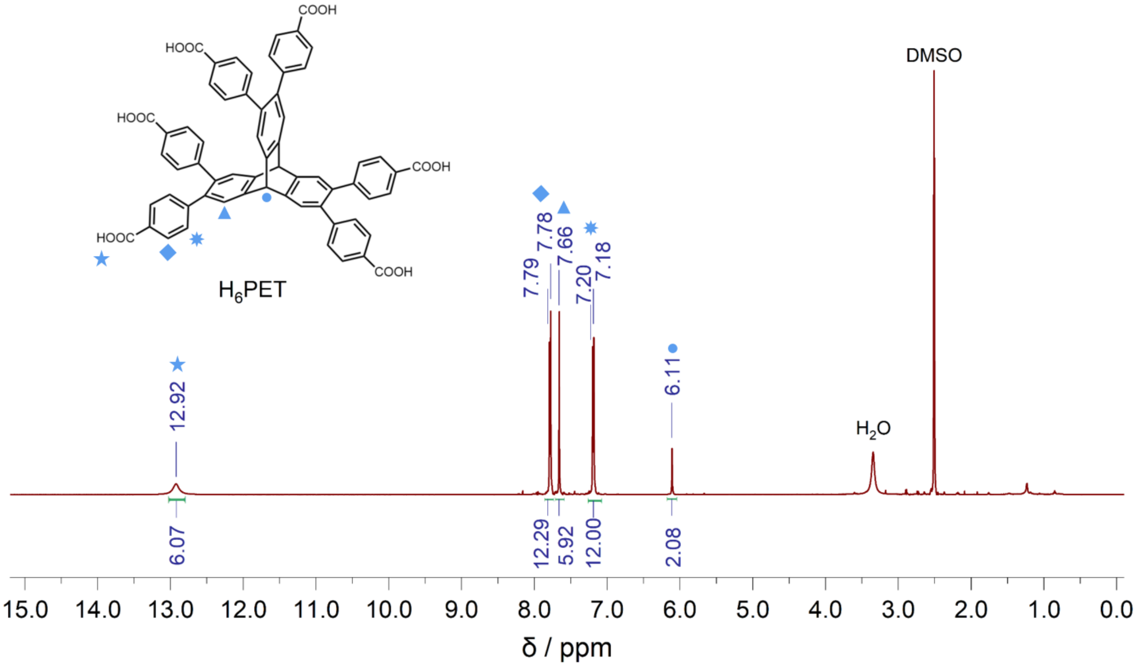

Figure S5. ^{1}H NMR Spectrum (500 MHz, DMSO-d_6, 298 K) of H_6PET.

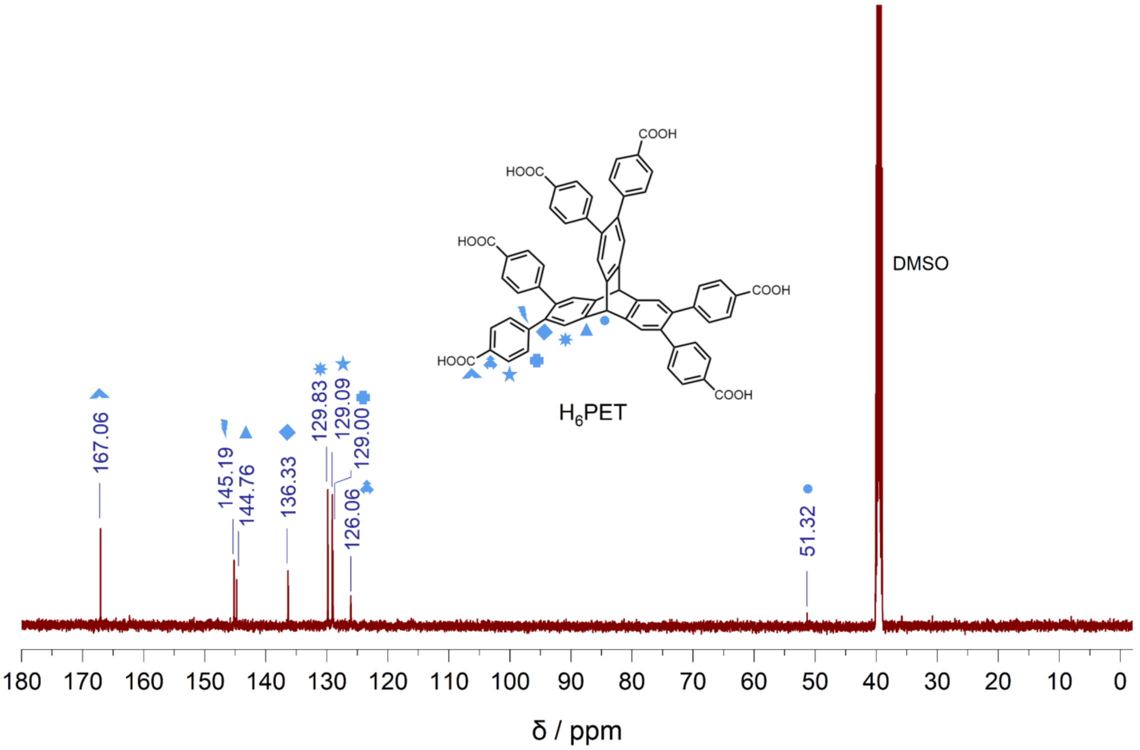

Figure S6. ^{13}C NMR Spectrum (126 MHz, DMSO-d$_6$, 298 K) of H$_6$PET.

Synthesis of HKU-9

A mixture of $(NH_4)_2Ce(NO_3)_6$ (34 mg, 0.06 mmol) and H$_6$PET (10 mg, 0.01 mmol) was added to a sample scintillation vial (20 ml). Then DMF (1.5 mL) and acetic acid (300 µL, 18 M) were sequentially added into the vial. After ultrasonicated, the sample vial was capped with aluminum foil lined screw cap, and placed in a preheated 100 °C oven for 24 h. The HKU-9 crystals were washed excessively with anhydrous DMF to remove the remaining starting materials. Solvent exchange *via* anhydrous acetone was conducted 3 times per day over 3 days.

Synthesis of HKU-90

HKU-9 crystals in acetone are dried in air. After 5 min, the phase transition completed forming HKU-90, which was confirmed with PXRD and SXRD.

Section S2.

Section S2.1. Powder X-ray diffraction patterns

As shown in Figure S7-S10, the experimental PXRD patterns measured with the zero-background sample holder and simulated PXRD patterns from the results of single crystal XRD match precisely. For the crystals soaked in acetone, they were measured on Rigaku

MiniFlex600 X-Ray Diffractometer equipped with Anton Paar BTS-500 stage at room temperature. The crystals were mounted on the sample holder (length × width × depth of the cavity: 16 × 14 × 0.2 mm), and covered with Kapton foils to prevent acetone from evaporation. The obtained PXRD pattern of HKU-9 in acetone was derived through the process of background subtraction.

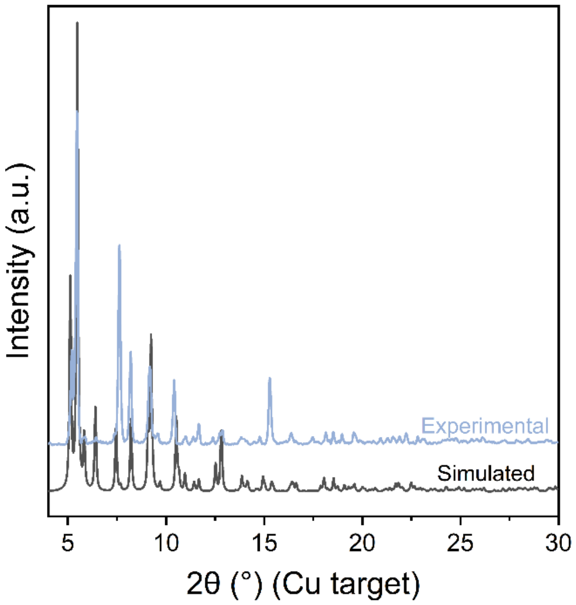

Figure S7. Experimental and simulated PXRD patterns of as-synthesized HKU-9.

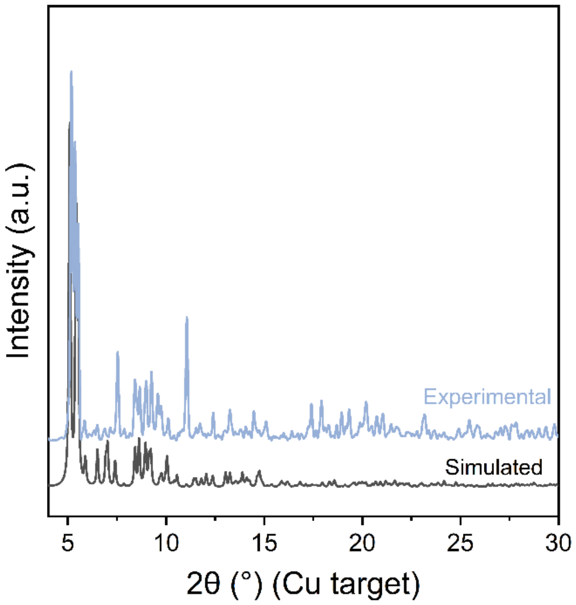

Figure S8. Experimental and simulated PXRD patterns of HKU-9 after DMF wash.

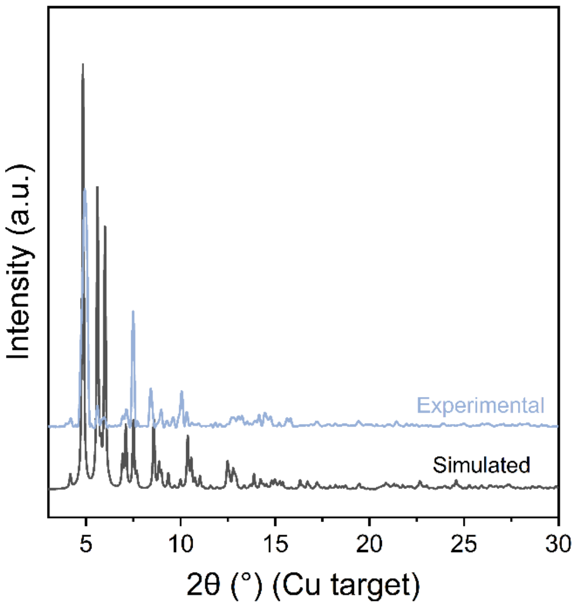

Figure S9. Experimental and simulated PXRD patterns of HKU-9 in acetone.

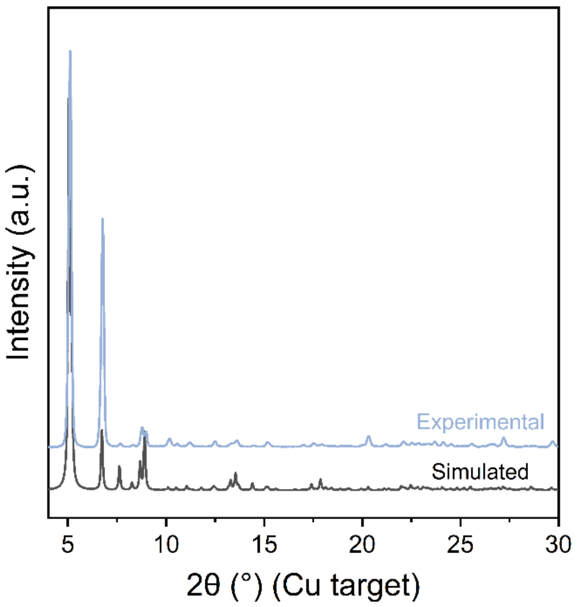

Figure S10. Experimental and simulated PXRD patterns of HKU-90.

Section S2.2. Reversibility test of phase transition from HKU-9 to HKU-90

To verify whether the phase transition process from HKU-9 to HKU-90 reversible, we soaked the HKU-90 crystals in acetone for three days and then solvent exchange with DMF for three days. As shown in Figure S11, powder X-ray diffraction (PXRD) patterns were collected after each step. The PXRD patterns of HKU-90, HKU-90 in acetone, and HKU-90 in DMF are nearly unchanged and different from HKU-9 in DMF and HKU-9 in acetone, implying that the phase transition process from HKU-9 to HKU-90 isn't reversible.

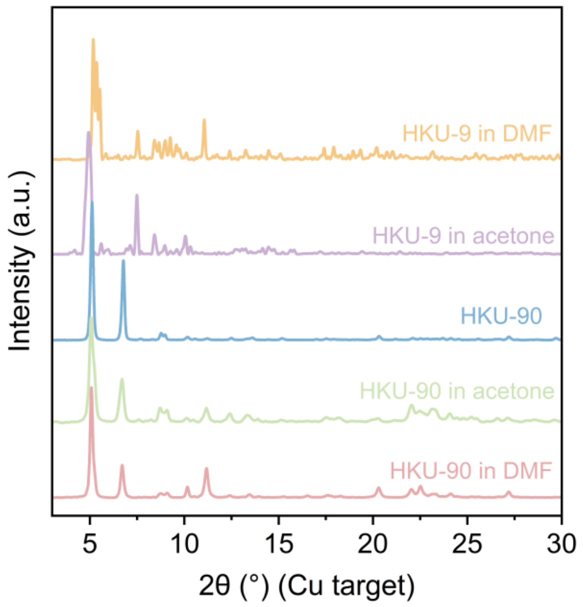

Figure S11. PXRD patterns of HKU-9 in DMF, HKU-9 in acetone, HKU-90 crystals, HKU-90 in acetone, and HKU-90 in DMF.

Section S2.3. Optical microscope images

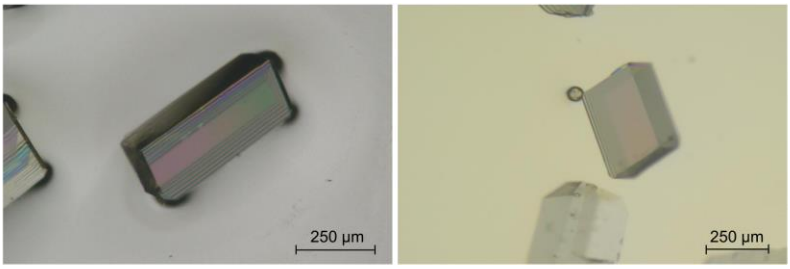

Figure S12. Optical images of as-synthesized HKU-9.

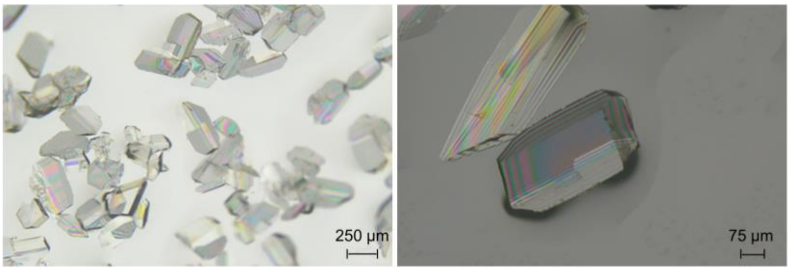

Figure S13. Optical images of HKU-9 in DMF.

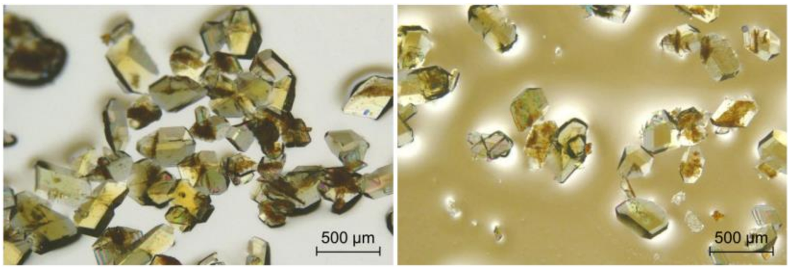

Figure S14. Optical images of HKU-9 in acetone.

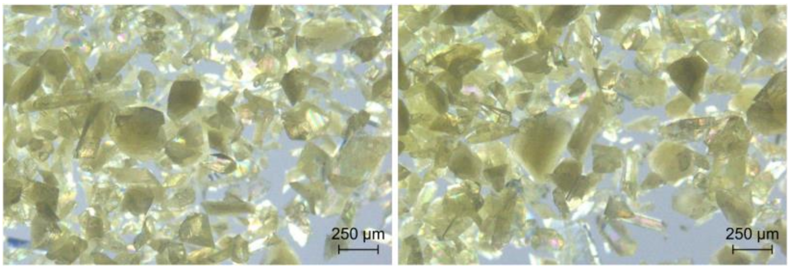

Figure S15. Optical images of HKU-90.

Section S2.4. X-ray photoelectron spectroscopy (XPS)

To understand more details of the chemical state of the phase transition, the XPS analysis[3] was displayed. From Figure S16a, the characteristic peaks of u' and u^0 located at 904.3 eV and 900.3 eV are assigned to Ce $3d_{3/2}$, the peaks of v' and v^0 at 885.7 eV and 881.9 eV belonged to Ce $3d_{5/2}$, in which u', u^0, v' and v^0 peaks correspond to Ce (III). After the phase transition to HKU-90, the chemical environment of Ce was unaffected as shown in Figure S16a. In contrast, the lattice oxygen (O_{lat}) of HKU-90 shift to high binging energy (531.3 eV) relative to the O_{lat} of HKU-9 (531.1 eV) in Figure S16b, demonstrating that the new forming bridged Ce-O-Ce bond changed the O chemical enviroment in HKU-90 after phase transition from HKU-9. In Figure S16b, other two peaks of O 1s spectra were fitted at 533.2 eV and 531.7 eV, corresponding to carboxyl group organic oxygen (O_{org}) and surface adsorbed oxygen (O_{ads}), respectively. As shown in Figure S16c, the C 1s spectra of HKU-9 have four diverse peaks corresponding to C=O, C−N, C-O and C-C with binding energies of 288.6 eV, 286.6 eV, 285.9 eV, 284.8 eV, respectively, in which the C−N bond was from DMF. In contrast, the C−N bond was not observed in HKU-90, indicating the absence of DMF in HKU-90.

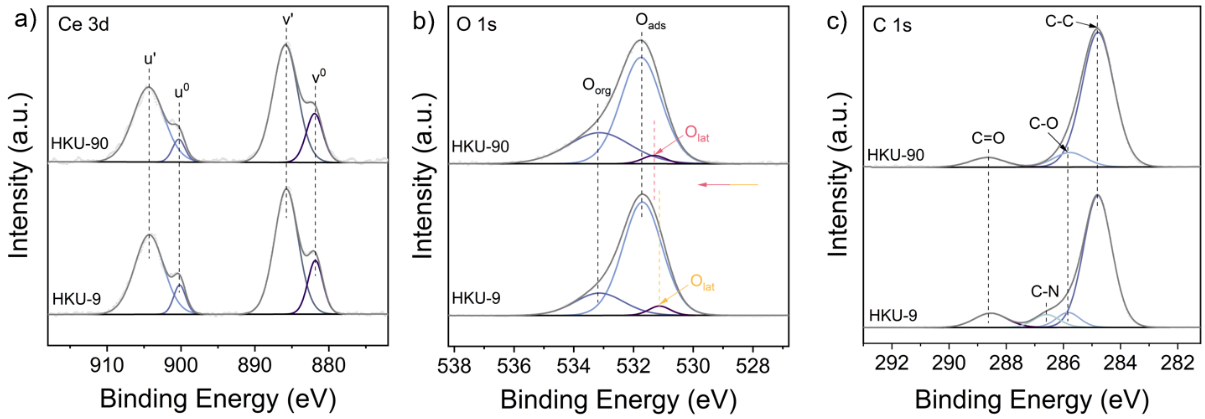

d)

HKU-9 / Elements	Atomic ratio from XPS (%)	Normalized atomic ratio from XPS
Ce 3d	1.77	2.00
C 1s	60.21	68.03

$C_{68}H_{50}N_2O_{16}Ce_2$
[$Ce_2PET(DMF)_2(H_2O)_2$]

e)

HKU-90 / Elements	Atomic ratio from XPS (%)	Normalized atomic ratio from XPS
Ce 3d	1.88	2.00
C 1s	58.61	62.35

$C_{62}H_{42}O_{17}Ce_2$
[$Ce_2(\mu\text{-}H_2O)PET(H_2O)_2\cdot 2H_2O$]

Figure S16. XPS spectra of HKU-9 and HKU-90: a) Ce 3d, b) O 1s, c) C 1s, respectively; Ratio of atomic by XPS analysis and comparison with the empirical formula from SXRD of **Table d)** HKU-9 and **Table e)** HKU-90. The atomic ratio from XPS (%) in the table was calculated from the normalized values of Ce (1.77%) to Ce (2.00%), based on the ratio of atomic measured data (%).

Additionally, we also obtained the ratio of atomic by XPS analysis. From Table S d-e, the measured ratios (%) of atomic of HKU-9 are Ce: 1.77, C: 60.21, O: 35.86, N: 2.16, and HKU-90 are Ce: 1.88, C: 58.61, O: 39.51, respectively. After calculated from the normalized values of Ce (1.77%) to Ce (2.00%) based on the ratio of atomic measured data (%), the ratios of atomic normalized data (%) (marked as atomic ratio from XPS) were consistent with the empirical formula from SXRD of HKU-9 and HKU-90, demonstrating the ratio of metal to ligand didn't change during phase transition. The higher ratio of O may be from the oxygen adsorbed on the surface of the samples.

Section S2.5. Thermogravimetric Analysis (TGA)

The TGA curve for the as-synthesized HKU-9 was shown in Figure S17. Prior to reaching 213 °C, the solvent encompassed within the crystal materials were vaporized and decomposed. After 213 °C, the decomposition of the crystal material commences, signifying the thermodynamic instability of the HKU-9. In contrast, the onset of decomposition for HKU-90 occurs at 368 °C (Figure S18), indicating that the crystal with linear SBU after the phase transition was more thermodynamically stable than HKU-9.

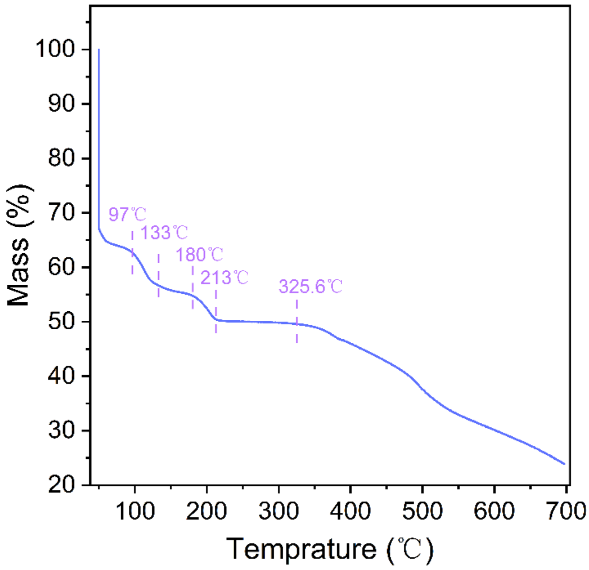

Figure S17. Thermal gravimetric analysis of as-synthesized HKU-9.

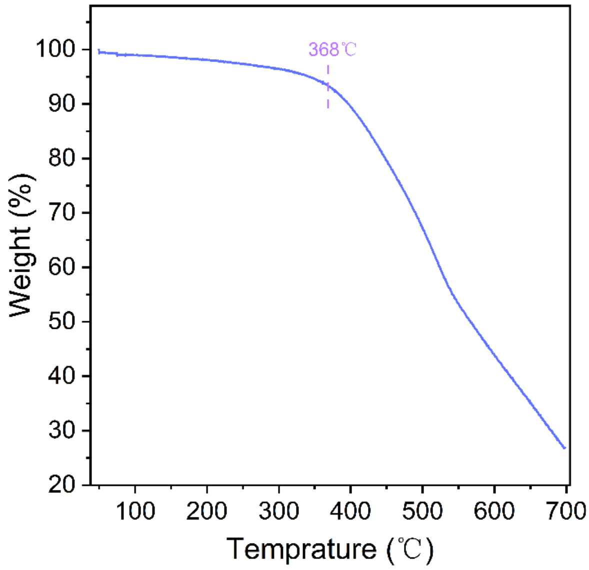

Figure S18. Thermal gravimetric analysis of HKU-90.

Section S2.6. Water stability test of HKU-9

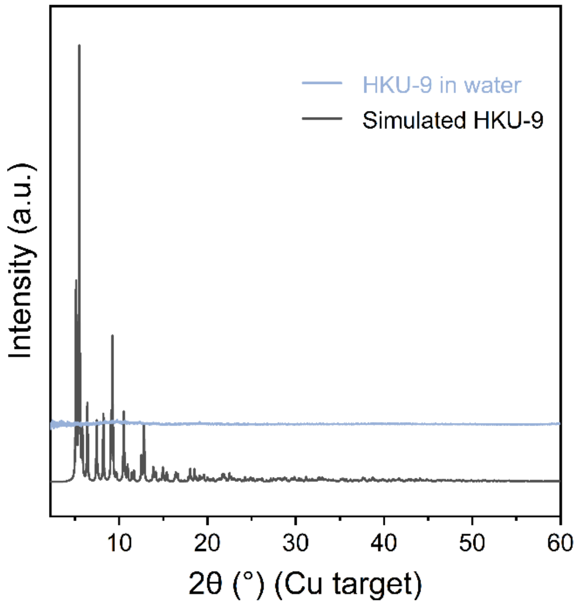

Figure S19. PXRD patterns of HKU-9 collected after exposure to water for 4 hours.

Section S3.

Section S3.1. Single crystal X-ray diffraction experimental conditions

The synchrotron SXRD data for as-synthesized HKU-9, in DMF, in acetone, and HKU-90 were collected at the Beamline BL17B1 and BL03HB at Shanghai Synchrotron Radiation Facility (SSRF, China). The parameters of collection were E = 18 keV, and a cryogenic temperature of 100 K was used. A crystal was coated with mineral oil and mounted on a 0.050 mm diameter loop. Space group determinations and .ins file generations were done using XPREP software with .hkl files generated from SADABS, and inspecting reconstruction images of the data frames. Initial models for refinements were generated by direct method implemented in SHELXS and refined with SHELXL,[4] which is implemented in Olex2.[5] Non-hydrogen atomic positions were assigned first. Metals in the structures were refined anisotropically followed by carbon and oxygen atoms. Hydrogen atoms were attached to the model by riding models. Residual electron densities in pores were assigned with carbon and oxygen, and refined to improve the framework models. When there is no meaningful improvement observed, the carbon and oxygen atoms in pores were removed, and the improved framework models were used for PLATON SQUEEZE.[6] The SQUEEZEd models were further refined with SHELXL until the parameters were converged.

Section S3.2. Single crystal data and structure refinement

Table S1. Single crystal data and structure refinement for as-synthesized HKU-9.

Identification code	HKU-9 assyn.
Empirical formula	$C_{34}H_{25}CeNO_8$
Formula weight/ (g/mol)	1,431.34
Temperature/K	100
Crystal system	monoclinic
Space group	*C*2/*c*
a/Å	19.062(5)
b/Å	34.512(8)
c/Å	24.047(6)
α/°	90
β/°	106.837(8)
γ/°	90
Volume/Å	15141.6(7)
Z	8
ρ_{calc} g/cm^3	0.628
μ/mm^{-1}	0.623
F(000)	2864.0
Crystal size/mm^3	0.1 × 0.2 × 0.2
Radiation	Synchrotron (λ= 0.6888)
2Θ range for data collection/°	2.686 to 52.862
Index ranges	$-23 \le h \le 23$, $-43 \le k \le 42$, $-30 \le 1 \le 29$
Reflections collected	51709
Independent reflections	15038 [R_{int} = 0.0749, R_{sigma} = 0.0698]
Data/restraints/parameters	15038/17/382
Goodness-of-fit on F^2	1.100
Final R indexes [I>=2σ (I)]	R_1 = 0.0932, wR_2 = 0.2679
Final R indexes [all data]	R_1 = 0.1068, wR_2 = 0.2912
Largest diff. peak/hole / e Å^{-3}	3.10/-1.99

Table S2. Single crystal data and structure refinement for HKU-9 in DMF.

Identification code	HKU-9 in DMF
Empirical formula	$C_{74}H_{60}N_4O_{16}Ce_2$
Formula weight/ (g/mol)	1455.5
Temperature/K	100
Crystal system	triclinic
Space group	*P*-1
a/Å	18.595(4)
b/Å	19.469(4)
c/Å	23.207(4)
α/°	93.853(6)
β/°	100.705(6)
γ/°	115.426(4)
Volume/Å	7353(2)
Z	2
ρ_{calc} g/cm^3	0.697
μ/mm^{-1}	0.649
F(000)	1553.0
Crystal size/mm^3	0.1 × 0.2 × 0.2
Radiation	Synchrotron (λ= 0.6888)
2Θ range for data collection/°	2.496 to 53.006
Index ranges	$-23 \leq h \leq 23$, $-24 \leq k \leq 24$, $-29 \leq 1 \leq 28$
Reflections collected	69596
Independent reflections	26356 [R_{int} = 0.0816, R_{sigma} = 0.1073]
Data/restraints/parameters	26356/6/717
Goodness-of-fit on F^2	1.036
Final R indexes [I>=2σ (I)]	R_1 = 0.1020, wR_2 = 0.2848
Final R indexes [all data]	R_1 = 0.1361, wR_2 =0.3203
Largest diff. peak/hole / e Å^{-3}	4.89/-1.98

Table S3. Single crystal data and structure refinement for HKU-9 in acetone.

Identification code	HKU9_inacetone
Empirical formula	$C_{148}H_{112}Ce_4O_{32}$
Formula weight/ (g/mol)	1511.5
Temperature/K	100
Crystal system	triclinic
Space group	*P*1
a/Å	17.118(3)
b/Å	19.664(3)
c/Å	21.949(4)
α/°	96.388(5)
β/°	99.671(6)
γ/°	108.601(5)
Volume/Å	6794(2)
Z	1
ρ_{calc} g/cm^3	0.724
μ/mm^{-1}	0.646
F(000)	1486.0
Crystal size/mm^3	0.1 × 0.2 × 0.2
Radiation	Synchrotron (λ = 0.6888)
2Θ range for data collection/°	2.492 to 50.998
Index ranges	$-21 \leq h \leq 21$, $-23 \leq k \leq 22$, $-27 \leq l \leq 27$
Reflections collected	80656
Independent reflections	46746 [R_{int} = 0.0589, R_{sigma} = 0.0969]
Data/restraints/parameters	46746/132/380
Goodness-of-fit on F^2	1.031
Final R indexes [I>=2σ (I)]	R_1 = 0.0966, wR_2 = 0.2453
Final R indexes [all data]	R_1 = 0.1420, wR_2 = 0.2960
Largest diff. peak/hole / e Å^{-3}	3.75/-1.79
Flack parameter	0.500(5)

Table S4. Single crystal data and structure refinement for HKU-90.

Identification code	HKU-90
Empirical formula	$C_{62}H_{42}O_{17}Ce_2$
Formula weight/ (g/mol)	1353
Temperature/K	100
Crystal system	monoclinic
Space group	*C*2/*c*
a/Å	20.951(2)
b/Å	34.073(4)
c/Å	16.2587(19)
α/°	90
β/°	103.683(6)
γ/°	90
Volume/Å	11277(2)
Z	4
ρ_{calc} g/cm^3	0.789
μ/mm^{-1}	0.838
F(000)	2659.0
Crystal size/mm^3	0.1 × 0.2 × 0.2
Radiation	Synchrotron (λ= 0.6888)
2Θ range for data collection/°	2.33 to 61.04
Index ranges	$-27 \leq h \leq 27$, $-37 \leq k \leq 39$, $-21 \leq l \leq 20$
Reflections collected	74998
Independent reflections	12857 [R_{int} = 0.0632, R_{sigma} = 0.0505]
Data/restraints/parameters	12857/0/370
Goodness-of-fit on F^2	1.030
Final R indexes [I>=2σ (I)]	R_1 = 0.0892, wR_2 = 0.2084
Final R indexes [all data]	R_1 = 0.1370, wR_2 = 0.2593
Largest diff. peak/hole / e Å^{-3}	5.63/-4.43

Section S4.

Section S4.1. N_2 isotherm measurements

The surface area and porosity of the materials were determined using N_2 adsorption isotherms at 77 K on a Micromeritics 3Flex Adsorption Analyzer. Before the experiments, around 0.1 g adsorbents were pre-degassed overnight at 333 K using the VacPrep 061 Sample Degas System. Subsequently, a further *in-situ* degassing at 363 K for 1 hour was performed to ensure the complete removal of water and other impurities.

Section S4.2. Various gas adsorption measurements

CO_2, C_2H_2, N_2O, CH_4 and N_2 adsorption isotherms were measured on a Micromeritics 3Flex Adsorption Analyzer. Prior to the measurements, an overnight degassing at 333 K and an *in situ* degassing at 363 K for 1 hour were conducted. The temperature of 273 K was achieved using water-ice bath. All gases employed in the experiments have a purity of 99.999%.

Section S4.3. DFT calculations for the phase transition

The quantum chemistry calculations were done with the ORCA 6.0.1 software.[7] The systems were fully optimized by DFT calculations using the hybrid functional PBE0, together with the large integration grid (DefGrid3) was used in all of the calculations. The molecular structures were extracted from X-ray data, since only the heavy atom positions were accurately determined, the heavy atom positions were frozen and the hydrogen atom positions were optimized. Both the Stuttgart large core pseudopotential and the all-electron 6-31G(d) basis sets were employed for the geometry optimization of the molecular structures. Whereas the MWB47 was used for the Ce atoms, and the 6-31G(d) was used for the C, O, and H atoms, respectively. The subsequent single point energy calculations were done with an all-electron triple-zeta valence basis set (def2-TZVP). The thermodynamics analysis and the interactions in bridge water and cerium atoms were conducted with Shermo,[8] VMD[9] and Multiwfn[10] software. Improve the simulation efficiency, the SBUs of HKU-9 and HKU-90 were intercepted for calculation, and the intercepted unsaturated bonds were filled with hydrogen bonds. Compared with the HKU-90, there is 5 H_2O molecules missing in the HKU-9, when doing the thermodynamics calculations, five more H_2O molecules energy will be considered.

The assessment of thermodynamic data (Table S5, obtained from quantum chemistry computations, led to the inference that the system's entropy experiences a notable reduction from 1418.7 kJ/mol/K to 518 kJ/mol/K during the phase transition from HKU-9 to HKU-90.

This convincingly demonstrates that the system's level of disarray substantially diminishes following the phase transition. Additionally, the system's enthalpy change is measured at -2120.5 kJ/mol, confirming that the phase transition is an exothermic process. Furthermore, the Gibbs free energy (△G) supports the conclusion that the reaction is spontaneous under standard temperature and pressure conditions, occurring without the necessity for external energy.

Table S5. Thermodynamic data and energy of the HKU-9 and HKU-90.

	Single point energy (kJ/mol)	H(T) (kJ/mol)	S (kJ/mol/K)	G(T) (kJ/mol)
HKU-9	-12549024.1	-12548168.0	475.0	-12548309.6
$5H_2O$	-1002255.7	-1001931.2	943.7	-1002212.6
HKU-90	-13553391.7	-13552219.8	518.9	-13552374.5
△	-2111.9	-2120.5		-1852.2

Upon analyzing the system's weak interactions via wave functions, it is postulated that bridging water contributes substantially to the system's behavior. As shown Figure S20 c, the diagram of the oxygen atom and the Ce atom in the bridging water bond is presented. The isosurface between the O and Ce is blue, which clearly demonstrates the presence of an electrostatic effect. In Figure S20 a-b, the Mulliken charges of Ce marked with yellow circle of HKU-90 (1.726) is larger than HKU-9 (1.435) in the system, which also support this point.

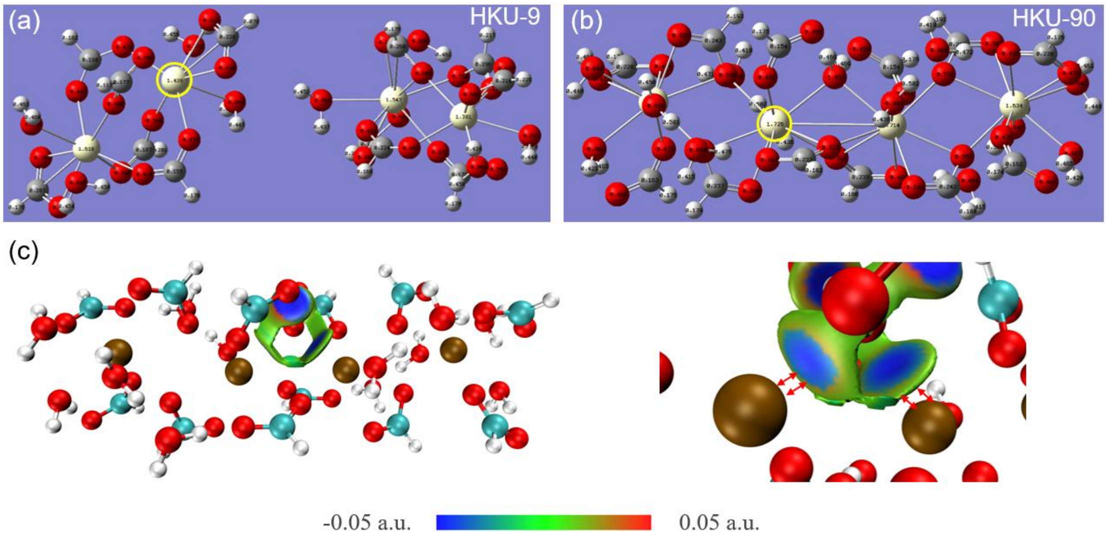

Figure S20. (a) and (b) The Mulliken charge distribution of HKU-9 and HKU-90. (c) Optimized structure and non-covalent interaction (NCI) map of HKU-90

Section S4.4. Heat of adsorption study

Considering the pore size of HKU-90 (> 10 Å), this pure-component selectivity should follow the equilibrium effect, where gases are adsorbed based on their different interactions with the adsorption sites. To further investigate the underlying adsorption mechanism, we tested the adsorption curves of C_2H_2 and CO_2 at different temperatures to calculate heat of adsorption (Figure S21 to Figure S24) The results shown in Figure S25 indicate that the zero-loading heat of adsorption for C_2H_2 is 24 kJ/mol, which is higher than that of CO_2 (19 kJ/mol). We believe this is attributed to the larger quadrupole moment of C_2H_2 than that of CO_2. The high C_2H_2 adsorption capacity is attributed to the highly porous structure in HKU-90, as evidenced by the high BET surface area.[11]

To evaluate the affinity of C_2H_2 and CO_2, the Virial equation was utilized to fit the combined isotherms at various temperatures. This approach aims to determine the heat of adsorption for C_2H_2 and CO_2 on HKU-9, as outlined below:

$$n(P) = ln(N) + \frac{1}{T}\sum_{i=0}^{m} a_i N^i + \sum_{i=0}^{n} b_i N^i$$

$$Q_{st} = -R\sum_{i=0}^{m} a_i N^i$$

In this equation, P and T denote pressure and temperature, and N is the amount adsorbed. The coefficients a_i and b_i are the virial coefficients, with m and n indicating the number of coefficients employed in the model fitting. R is the universal gas constant, and Q_{st} represents the coverage-dependent heat of adsorption. Corresponding discussions are marked in yellow and added to the revised supporting information. Please see "Section S4." in revised supporting information.

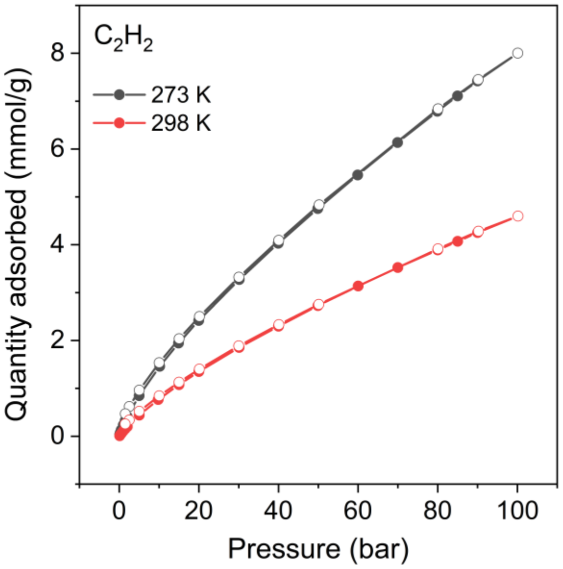

Figure S21. C_2H_2 adsorption isotherms on HKU-90 measured at 273 K and 298 K.

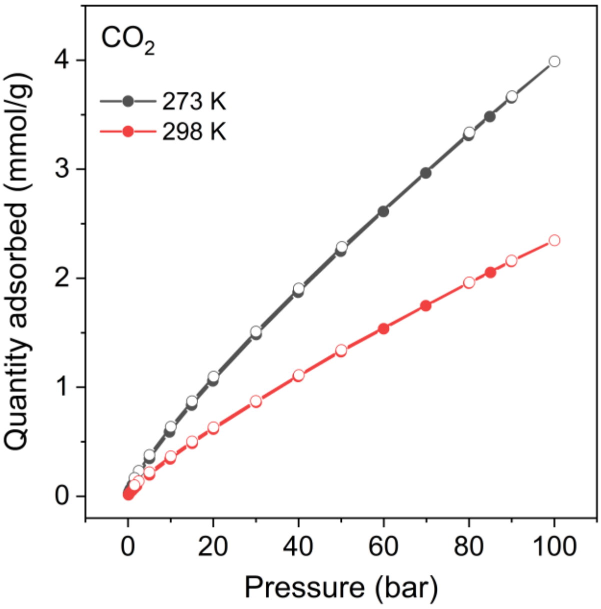

Figure S22. CO_2 adsorption isotherms on HKU-90 measured at 273 K and 298 K.

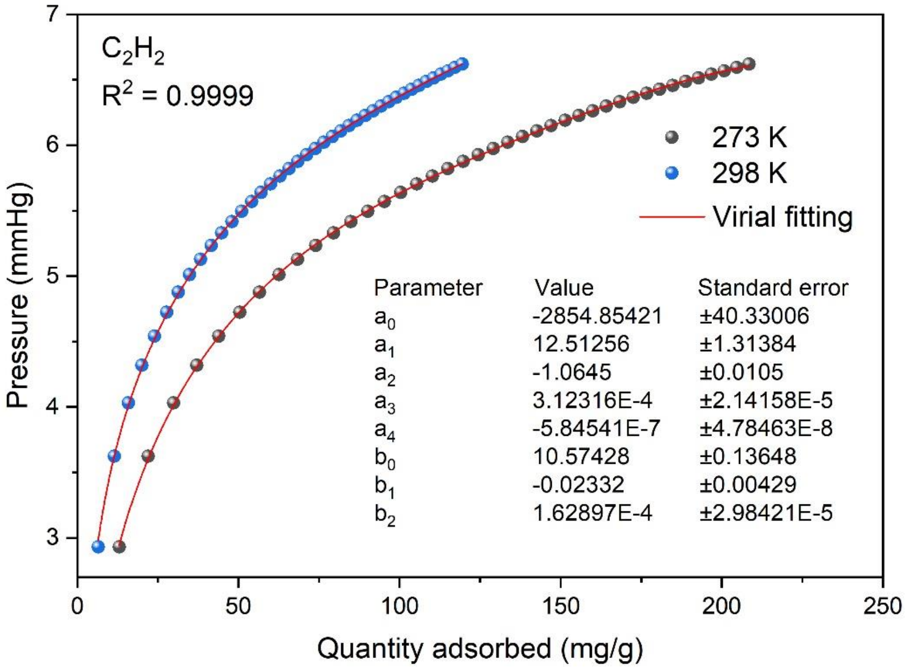

Figure S23. Fitting parameters by virial equation of C_2H_2 on HKU-90 at 273 K and 298 K.

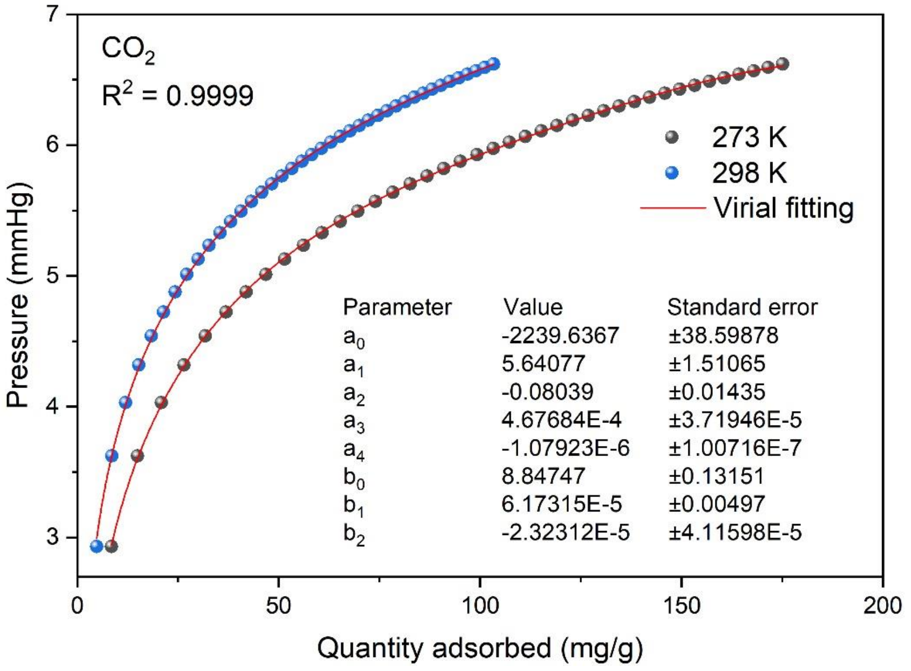

Figure S24. Fitting parameters by virial equation of CO_2 on HKU-90 at 273 K and 298 K.

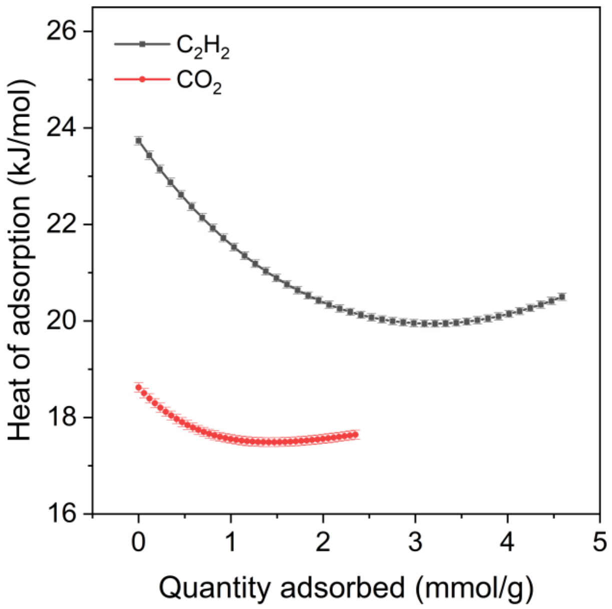

Figure S25. Heat of adsorption of C_2H_2 and CO_2 on HKU-90.

Table S6. Textural parameter comparison of Ce-based MOFs.

Materials	BET surface area (m^2/g)	Pore volume (cm^3/g)	Average pore size (Å)
CeO_2-MOF/350[12]	117	0.30	88.0
CeO_2-MOF/500	73	0.30	189.0
CuCe-BTC[13]	133	0.14	33.0
MOF(Zr)-MOF(Ce/La)[14]	332	0.25	30.2
$CeL(H_2O)_2$[15]	467	0.20	-
Ce-BTC-1[16]	481	0.38	41.5
Ce-BDC-1	1286	0.52	20.2
Ce_TzTz[17]	1136	0.89	15.2
Ce_TzTz_PyPy	238	0.57	14.0
$Ce\text{-}BTC_{250}$[18]	648	0.38	86.0
Ce-UiO-66[19]	984	0.43	11.0
Ce-UiO-66-NH_2	944	0.42	9.9
Ce-UiO-66-BPyDC[20]	2120	0.85	-
Ce-MOF-808	1725	0.62	-
Ce-MOF-808-1.5-80FA[21]	2077	-	19.0
Ce-MOF-808-1-80FA	1106	-	19.0
Ce-MOF-808-3-80FA	612	-	19.0
HKU-90 (This work)	2660	1.08	11.0

Table S7. Comparison of adsorbents for C_2H_2/CO_2 (50/50, v/v) separation at 273 K.

Materials	C_2H_2 capacity (mmol/g)[a]	CO_2 capacity (mmol/g)[a]	C_2H_2/CO_2 selectivity[b]
DICRO-4-Ni-i[22]	1.77	0.96	1.844
ZJU-60a[23]	7.79	4.23	1.842
NKMOF-1-Ni[24]	2.91	2.78	1.045
SNNU-45[25]	8.62	7.46	1.155
TIFSIX-2-Cu-i[26]	4.64	5.74	0.808
TIFSIX-2-Ni-i	3.59	3.08	1.166
PCP-33[27]	8.07	4.70	1.717
FeNi-M'MOF[28]	4.66	3.25	1.434
UTSA-74[29]	5.92	3.88	1.526
UPC-200(Al)-F[30]	9.52	4.66	2.043
UPC-200(Cr)-F	7.22	3.63	1.989
SIFSIX-dps-Cu[31]	4.99	2.30	2.169
FJU-90[32]	9.59	7.29	1.316
ATC-Cu[33]	6.01	4.72	1.273
FJU-6-TATB[34]	4.49	2.37	1.895
HKU-90 (This work)	8.22	3.80	2.163

[a] Gas capacity was measured at 100 kPa.
[b] Gas selectivity was determined by the ratio of the adsorption capacity of the two gases at 100 kPa.
[c] The data were collected at 298 K.

References

(1) Norvez, S., Liquid crystalline triptycene derivatives. *J. Org. Chem.* **1993,** *58,* 2414-2418.
(2) Li, P.; Li, P.; Ryder, M. R.; Liu, Z.; Stern, C. L.; Farha, O. K.; Stoddart, J. F., Interpenetration Isomerism in Triptycene-Based Hydrogen-Bonded Organic Frameworks. *Angew. Chem. Int. Ed.* **2019,** *58,* 1664-1669.
(3) Heide, P., Appendix B: Binding Energies (B.E.XPS or B.E.XRF) of the Elements. In X-Ray Photoelectron Spectroscopy, (John Wiley & Sons, **2011,** pp 171-176).
(4) Sheldrick, G., Crystal structure refinement with SHELXL. *Acta Crystallogr. C* **2015,** *71,* 3-8.
(5) Dolomanov, O. V.; Bourhis, L. J.; Gildea, R. J.; Howard, J. A. K.; Puschmann, H., OLEX2: a complete structure solution, refinement and analysis program. *J. Appl. Crystallogr.* **2009,** *42,* 339-341.
(6) Spek, A., PLATON SQUEEZE: a tool for the calculation of the disordered solvent contribution to the calculated structure factors. *Acta Crystallogr. C* **2015,** *71,* 9-18.
(7) Neese, F., Software update: The ORCA program system—Version 5.0. WIREs Computational Molecular Science **2022,** *12,* e1606.
(8) Lu, T.; Chen, Q., Shermo: A general code for calculating molecular thermochemistry properties. *Comput. Theor. Chem.* **2021,** *1200,* 113249.
(9) Humphrey, W.; Dalke, A.; Schulten, K., VMD: Visual molecular dynamics. *J. Mol. Graph* **1996,** *14,* 33-38.
(10) Lu, T., A comprehensive electron wavefunction analysis toolbox for chemists, Multiwfn. *J. Chem. Phys.* **2024,** *161,* 082503.

(11) Yang, R.T. Adsorbents: fundamentals and applications. (John Wiley & Sons, **2003**).
(12) Chen, X.; Yu, E.; Cai, S.; Jia, H.; Chen, J.; Liang, P., In situ pyrolysis of Ce-MOF to prepare CeO_2 catalyst with obviously improved catalytic performance for toluene combustion. *Chem. Eng. J.* **2018,** *344,* 469-479.
(13) Jampaiah, D.; Shah, D.; Chalkidis, A.; Saini, P.; Babarao, R.; Arandiyan, H.; Bhargava, S. K., Bimetallic Copper–Cerium-Based Metal–Organic Frameworks for Selective Carbon Dioxide Capture. *Langmuir* **2024,** *40,* 9732-9740.
(14) Liu, R.; Song, J.; Zhao, J.; Wang, Z.; Xu, J.; Yang, W.; Hu, J., Novel MOF (Zr)–on-MOF (Ce/La) adsorbent for efficient fluoride and phosphate removal. *Chem. Eng. J.* **2024,** *497,* 154780.
(15) Bejan, D.; Bahrin, L. G.; Shova, S.; Marangoci, N. L.; Kökçam-Demir, Ü.; Lozan, V.; Janiak, C., New microporous lanthanide organic frameworks. Synthesis, structure, luminescence, sorption, and catalytic acylation of 2-naphthol. *Molecules* **2020,** *25,* 3055.
(16) He, J.; Pei, C.; Yang, Y.; Lai, B.; Sun, Y.; Yang, L., The structural design and valence state control of cerium-based metal-organic frameworks for their highly efficient phosphate removal. *J. Clean. Prod.* **2021,** *321,* 128778.
(17) Pugliesi, M.; Cavallo, M.; Atzori, C.; Garetto, B.; Borfecchia, E.; Donà, L.; Civalleri, B.; Tuci, G.; Giambastiani, G.; Galli, S., Selective Carbon Dioxide versus Nitrous Oxide Adsorption in Cerium (IV) Bithiazole and Bipyridyl Metal-Organic Frameworks. *Adv. Funct. Mater.* **2024,** *34,* 2403017.
(18) Zhang, X.; Hou, F.; Li, H.; Yang, Y.; Wang, Y.; Liu, N.; Yang, Y., A strawsheave-like metal organic framework Ce-BTC derivative containing high specific surface area for improving the catalytic activity of CO oxidation reaction. *Micropor. Mesopor. Mater.* **2018,** *259,* 211-219.
(19) Senith Ravishan Fernando, J.; Asaithambi, S. S.; Maruti Chavan, S., Amino-Functionalizing Ce-Based MOF UiO-66 for Enhanced CO_2 Adsorption and Selectivity. *ChemPlusChem* **2024,** *89,* e202400107.
(20) Lammert, M.; Glißmann, C.; Reinsch, H.; Stock, N., Synthesis and characterization of new Ce (IV)-MOFs exhibiting various framework topologies. *Cryst. Growth Des.* **2017,** *17,* 1125-1131.
(21) Yassin, J. M.; Taddesse, A. M.; Sánchez-Sánchez, M., Room temperature synthesis of high-quality Ce (IV)-based MOFs in water. *Micropor. Mesopor. Mater.* **2021,** *324,* 111303.
(22) Scott, H. S.; Shivanna, M.; Bajpai, A.; Madden, D. G.; Chen, K.-J.; Pham, T.; Forrest, K. A.; Hogan, A.; Space, B.; Perry IV, J. J., Highly selective separation of C_2H_2 from CO_2 by a new dichromate-based hybrid ultramicroporous material. *ACS Appl. Mater. Interfaces* **2017,** *9,* 33395-33400.
(23) Duan, X.; Zhang, Q.; Cai, J.; Yang, Y.; Cui, Y.; He, Y.; Wu, C.; Krishna, R.; Chen, B.; Qian, G., A new metal–organic framework with potential for adsorptive separation of methane from carbon dioxide, acetylene, ethylene, and ethane established by simulated breakthrough experiments. *J. Mater. Chem. A* **2014,** *2,* 2628-2633.
(24) Peng, Y. L.; Pham, T.; Li, P.; Wang, T.; Chen, Y.; Chen, K. J.; Forrest, K. A.; Space, B.; Cheng, P.; Zaworotko, M. J., Robust ultramicroporous metal–organic frameworks with benchmark affinity for acetylene. *Angew. Chem. Int. Ed.* **2018,** *57,* 10971-10975.
(25) Li, Y. P.; Wang, Y.; Xue, Y. Y.; Li, H. P.; Zhai, Q. G.; Li, S. N.; Jiang, Y. C.; Hu, M. C.; Bu, X., Ultramicroporous building units as a path to Bi-microporous metal–organic frameworks with high acetylene storage and separation performance. *Angew. Chem. Int. Ed.* **2019,** *131,* 13724-13729.
(26) Chen, K.-J.; Scott, H. S.; Madden, D. G.; Pham, T.; Kumar, A.; Bajpai, A.; Lusi, M.; Forrest, K. A.; Space, B.; Perry, J. J., Benchmark C_2H_2/CO_2 and CO_2/C_2H_2 separation by two closely related hybrid ultramicroporous materials. *Chem* **2016,** *1,* 753-765.
(27) Duan, J.; Jin, W.; Krishna, R., Natural gas purification using a porous coordination polymer with water and chemical stability. *Inorg. Chem.* **2015,** *54,* 4279-4284.
(28) Gao, J.; Qian, X.; Lin, R. B.; Krishna, R.; Wu, H.; Zhou, W.; Chen, B., Mixed metal–organic framework with multiple binding sites for efficient C_2H_2/CO_2 separation. *Angew. Chem. Int. Ed.* **2020,** *59,* 4396-4400.
(29) Luo, F.; Yan, C.; Dang, L.; Krishna, R.; Zhou, W.; Wu, H.; Dong, X.; Han, Y.; Hu, T.-L.; O'Keeffe, M., UTSA-74: a MOF-74 isomer with two accessible binding sites per metal center for highly selective gas separation. *J. Am. Chem. Soc.* **2016,** *138,* 5678-5684.

(30) Fan, W.; Yuan, S.; Wang, W.; Feng, L.; Liu, X.; Zhang, X.; Wang, X.; Kang, Z.; Dai, F.; Yuan, D., Optimizing multivariate metal–organic frameworks for efficient C_2H_2/CO_2 separation. *J. Am. Chem. Soc.* **2020,** *142*, 8728-8737.
(31) Wang, J.; Zhang, Y.; Su, Y.; Liu, X.; Zhang, P.; Lin, R.-B.; Chen, S.; Deng, Q.; Zeng, Z.; Deng, S., Fine pore engineering in a series of isoreticular metal-organic frameworks for efficient C_2H_2/CO_2 separation. *Nat. Commun.* **2022,** *13*, 200.
(32) Ye, Y.; Ma, Z.; Lin, R.-B.; Krishna, R.; Zhou, W.; Lin, Q.; Zhang, Z.; Xiang, S.; Chen, B., Pore space partition within a metal–organic framework for highly efficient C_2H_2/CO_2 separation. *J. Am. Chem. Soc.* **2019,** *141*, 4130-4136.
(33) Niu, Z.; Cui, X.; Pham, T.; Verma, G.; Lan, P. C.; Shan, C.; Xing, H.; Forrest, K. A.; Suepaul, S.; Space, B., A MOF-based ultra-strong acetylene nano-trap for highly efficient C_2H_2/CO_2 separation. *Angew. Chem. Int. Ed.* **2021,** *133*, 5343-5348.
(34) Liu, L.; Yao, Z.; Ye, Y.; Yang, Y.; Lin, Q.; Zhang, Z.; O'Keeffe, M.; Xiang, S., Integrating the pillared-layer strategy and pore-space partition method to construct multicomponent MOFs for C_2H_2/CO_2 separation. *J. Am. Chem. Soc.* **2020,** *142*, 9258-9266.
